# The frequency of maternal morbidity: A systematic review of systematic reviews

**DOI:** 10.1002/ijgo.12468

**Published:** 2018-05-23

**Authors:** Giorgia Gon, Andreia Leite, Clara Calvert, Susannah Woodd, Wendy J. Graham, Veronique Filippi

**Affiliations:** ^1^ Department of Infectious Disease Epidemiology London School of Hygiene and Tropical Medicine London UK

**Keywords:** Frequency, Incidence, Maternal health, Morbidity, Prevalence, Systematic review

## Abstract

**Background:**

Estimates of the burden of maternal morbidity are patchy.

**Objective:**

To conduct a systematic review of systematic reviews of maternal conditions to: (1) make available the most up‐to‐date frequency estimates; (2) identify which conditions do not have reliable estimates; and (3) scrutinize the quality of the available reviews.

**Search strategy:**

We searched Embase, MEDLINE, and CINAHL, combining terms for pregnancy, frequency (e.g. prevalence, incidence), publication type, and specific terms for each of 121 conditions.

**Selection criteria:**

We included peer‐reviewed systematic reviews aiming to estimate the frequency of at least one of the conditions in WHO's list of maternal morbidities, with estimates from at least two countries.

**Data collection and analysis:**

We present the frequency estimates with their uncertainty bounds by condition, region, and pregnancy/postpartum period. We also assess and present information on the quality of the systematic reviews.

**Main results:**

Out of 11 930 found, 48 reviews were selected and one more was added. From 49 reviews we extracted 34 direct and 60 indirect frequency estimates covering 35 conditions. No review was available for 71% of the conditions on the WHO list. The extracted estimates show substantial maternal morbidity, spanning the time before and beyond childbirth. There were several gaps in the quality of the reviews. Notably, one‐third of the estimates were based only on facility‐based studies.

**Conclusions:**

Good‐quality systematic reviews are needed for several conditions, as a research priority.

## Abstract 2

This study highlights that substantial maternal morbidity occurs along the pregnancy–postpartum continuum, and that conducting quality systematic reviews is a priority research gap.

## 1 INTRODUCTION

The Global Burden of Disease (GBD) study group estimated that in 2013 alone, maternal conditions contributed to 18 027 800 disability‐adjusted life years, including morbidity from hemorrhage, infection, hypertension, abortion complications, obstructed labor, late and indirect maternal deaths, and those deaths aggravated by HIV.^1^ A recent publication suggested that the five main direct obstetric causes of morbidity resulted in 27 million morbid episodes in 2015.^2^ These sources, however, underestimate the true burden of disease attributable to pregnancy‐related conditions as they include only a few maternal conditions.^3^ They ignore common conditions, such as postpartum depression,^3^ and mild but prevalent conditions, such as urinary incontinence that affects over one‐third of the pregnant population in Europe alone.^4^

The WHO recently published a comprehensive list of maternal morbidities, comprising 121 direct and indirect conditions.^5^ This list provides an important framework to understand what conditions constitute maternal morbidity, although the extent to which each of the listed morbidities contributes to the total burden remains unclear.^3^ Addressing this gap in our knowledge is necessary to better prioritize conditions for intervention. Furthermore, identifying the conditions that we know the least about is also important so they can be included in the future research agenda.^2^

The aim of this systematic review is to identify existing systematic reviews quantifying the burden of each of the conditions identified in WHO's list of maternal morbidities. Compiling this information will enable us to: (1) make available the most up‐to‐date frequency (e.g. prevalence, incidence) estimates on each maternal condition; (2) identify which conditions lack reliable estimates; and (3) discuss the quality of the systematic reviews and the reliability of the available estimates.

## 2 MATERIALS AND METHODS

### 2.1 Search strategy

We conducted a systematic search in Embase, MEDLINE, and the Cumulative Index to Nursing and Allied Health Literature (CINAHL) using a combination of free text terms and Medical Subject Headings (MeSH terms). We combined terms for the following domains: pregnancy (e.g. maternal, antenatal), frequency of the disease (e.g. prevalence, incidence), publication type (e.g. systematic review, meta‐analysis), and specific terms for each of the 121 conditions described in the WHO maternal morbidity list by Chou et al.^5^ The search strategy was prepared by AL, CC, and GG, with input from VF, SW, and an experienced librarian. The complete strategy is provided in Appendix S1. The search was restricted to humans and there were no language restrictions. The search was last run on July 23, 2016. In addition, we included further relevant systematic reviews known to the authors of this paper but not identified by the search, and we searched the reference lists of eligible studies. We used the MOOSE guidelines for conducting systematic reviews of observational studies to carry out and report on this review.^6^

### 2.2 Inclusion and exclusion criteria for selection of systematic reviews

We included peer‐reviewed systematic reviews that aimed to estimate the frequency of at least one of the maternal conditions listed in Chou et al.^5^ and which included estimates from at least two countries. The latter was a way to ensure we included estimates representing a region rather than a specific country. We included systematic reviews that included at least one paper published in or after 2006, as an attempt to provide recent estimates.

We excluded papers that: (1) did not mention frequency of the outcome among pregnant women in the abstract; (2) only reviewed studies for certain subgroups (e.g. rural women, or women with a specific health condition, women giving birth to twins, or women with a previous cesarean delivery); (3) focused only on risk factors or consequences of a certain maternal condition; (4) were not systematic reviews; (5) primarily included interventions for or investigated the effect of a single individual characteristic of the relevant maternal conditions; (6) were not possible to access in full; and (7) provided insufficient information on their inclusion and exclusion criteria in the text and the authors did not provide this information after we had attempted to contact them twice.

### 2.3 Data extraction

Data extraction from eligible reviews was carried out at two levels: (1) information on the overall paper; and (2) the specific frequency estimate. We did not extract information from the primary studies included in the eligible systematic reviews. For the study selection of the systematic reviews, one author (AL) screened titles and abstracts, and 10% of these were also screened by GG.

During the first level of data extraction, either AL, GG, or CC extracted data from eligible reviews such as the region reviewed, the databases searched, and the inclusion criteria applied for study selection. We also extracted detailed information on the quality of the systematic reviews. This was performed by AL with double extraction of 50% of the reviews (by GG). To assess the quality, we adapted the quality assessment tool for assessing systematic reviews proposed by Mann et al.,^7^ which is a modified version of the Overview Quality Assessment Questionnaire (OQAQ). Our adaptations included a question on whether authors clearly specified the source of the data—whether hospital, population, or unknown—and whether the search strategy was clearly laid out. The details on the modified OQAQ tool we used (which included 13 criteria) and the way we scored against it are available in Appendix S2. We did not provide numerical summary quality scores for each eligible review because these could mask the relative importance of the different quality indicators. Instead, we used a traffic light system, and we calculated the overall proportion of articles scoring a specific color (e.g. green) for each question.

For the second level of data extraction, either GG or AL extracted the frequency estimates together with information on whether these were population‐ or facility‐based, the denominator for each, the countries they represented, the type of estimate (i.e. incidence or prevalence), and the diagnostic tools used for case ascertainment. Estimates were classified as population based if: (1) authors clearly said they were population based, or (2) the sample was recruited from facilities in countries where virtually all deliveries happen in facilities. For 50% of the papers included, we carried out double extraction of the frequency estimates and their details. Any discrepancies were resolved through discussion.

To select “the best” estimates to extract from systematic reviews where several frequency estimates were presented, we established the following rules that were applied in hierarchical order:

1. Select population‐based estimates over (a) facility‐based estimates, or (b) estimates combining both facility‐ and population‐based estimates.
2. When both pooled estimates (i.e. a weighted average) and the range of estimates from individual studies were provided, extract the pooled estimate.
3. Select estimates covering the widest geographical area.
4. Select the most recent estimates, in terms of data capture period.

For example, if a review reported a frequency estimate based on community‐based studies and a separate estimate for facility‐based studies, then we only extracted the estimate based on the community‐based studies. If a study reported weighted means based only on facility‐based studies separately for West Africa and for the whole of the African continent, then we selected only the estimate for Africa.

Estimates from systematic reviews where only a single study was identified are equivalent to reporting estimates from a single primary study; therefore, for the purposes of reporting estimates of maternal morbidity, we did not include estimates from such systematic reviews. For any reviews that were eligible based on our inclusion and exclusion criteria, but from which we could not extract a frequency estimate, we have included the paper in the main description but we do not report an estimate from it.

### 2.4 Analysis

We transformed all estimates into percentages for presentation and comparison. For a particular review and each condition reported on, we present the frequency and the type of estimate (prevalence or incidence), the uncertainty range, region, pregnancy period, diagnostic tools, and data source (facility‐ vs population‐based). We report the region (or group of countries) for each estimate based on the countries covered by the primary studies included in the systematic review, which underpin each estimate. For those conditions reported by multiple eligible systematic reviews, we present estimates from each of those reviews. If those reviews reporting on the same condition included some of the same primary studies, we did not choose between the reviews because each review had distinct inclusion/exclusion criteria; for example, some reviews focused on certain countries or study designs. If a systematic review reported on multiple conditions of interest, we extracted estimates for each of these conditions.

## 3 RESULTS

**Figure 1:**
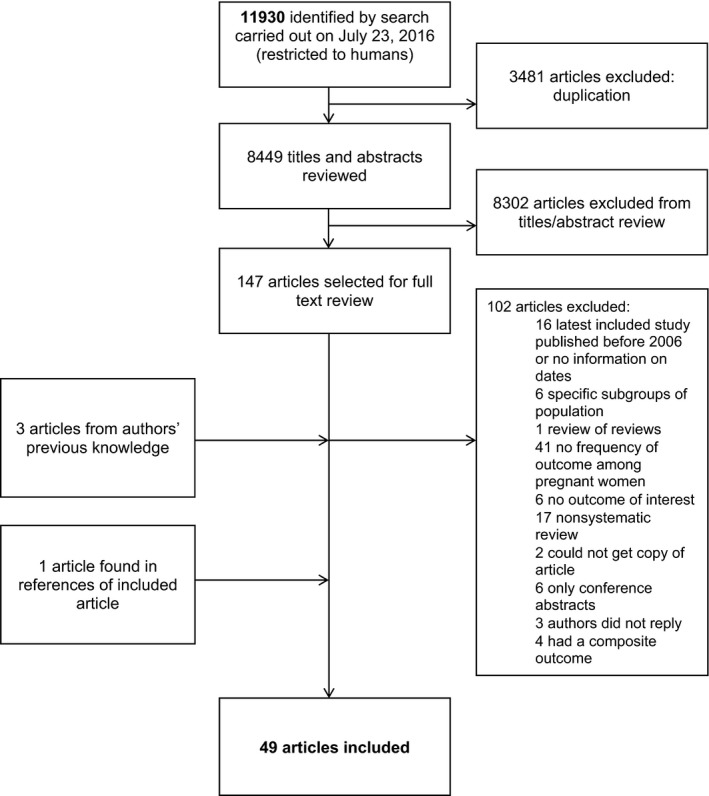
Study selection for inclusion in the systematic review.

We identified 11 930 results from searches across Embase, MEDLINE, and CINAHL, of which 3481 were duplicates and 8302 were unrelated to the topic of interest after screening the title and abstract (Fig. 1). A total of 150 papers were selected for full‐text review, of which three were added to the search results based on our previous knowledge. Full‐text review led to the exclusion of 102 papers for the reasons stated in Figure 1, including four articles that were excluded because they only reported composite outcomes, aggregating the frequency of multiple conditions in the WHO list.^8^, ^9^, ^10^, ^11^ We selected 48 eligible systematic reviews, and from searching their references we found one more. From these 49 eligible reviews, we extracted 34 direct and 60 indirect frequency estimates covering 35 conditions. The full list of included papers is provided in Appendix S3.

### 3.1 Availability of systematic reviews

We found that for 71% of conditions in the WHO list by Chou et al.^5^ there was no systematic review available (Appendix S3). The systematic reviews we found covered a substantial proportion (36%) of direct and coincidental maternal conditions, as well as several mental disorders (63%), and maternal infectious and parasitic diseases (46%). Under the direct morbidity umbrella, our search did not yield any systematic review for three categories: (1) pregnancy‐related infection, such as puerperal sepsis or mastitis; (2) cardiovascular obstetric complications such as peripartum cardiomyopathy; and (3) complications related to anesthesia.

In addition, we did not find any eligible systematic reviews for nine indirect morbidity categories listed by Chou et al.,^5^ as outlined in full in Appendix S3. For example, none were found in the category called “Other maternal diseases classifiable elsewhere but complicating pregnancy, childbirth and the puerperium,” which includes anemia, and we also found none under “Diseases of the musculoskeletal system and connective tissue,” including back pain. A systematic review on anemia was not considered eligible because it aimed to review primary studies investigating the risk factors for anemia, therefore excluding studies that investigated the frequency of anemia but did not report on effect‐size estimates of risk factors.^12^

Some conditions had multiple available systematic reviews. The highest number was identified for gestational diabetes (eight systematic reviews),^13^, ^14^, ^15^, ^16^, ^17^, ^18^, ^19^, ^20^ followed by infectious hepatitis, intimate partner violence, and postpartum depression, with four systematic reviews each. Although two systematic reviews were eligible and included, we have not reported frequency estimates for these because either the estimates were based on only one study per condition,^21^ or they reviewed a variety of conditions and denominators that were difficult to combine to present summary estimates here^22^; details are provided in Appendix S3.

### 3.2 Characteristics of available systematic reviews

Details of the 94 frequency estimates extracted, including the denominator and the geographical area for each are presented by pregnancy period and by type of estimate in Table 1 (direct maternal morbidities) and Table 2 (indirect maternal morbidities). The systematic reviews used several types of prevalence (n=77) or incidence (n=17) estimates, including ranges, weighted means, crude means, and medians. The denominator varied according to the condition and the authors, and generally included births, pregnancies, deliveries, women of reproductive age (for fistula), and person‐years at risk (for HIV). Further details about the estimates are in Tables 1 and 2, and additional details are provided in Appendix S4.

**Table 1:** Direct maternal morbidity estimates

Condition	Author	Group of countries or region	Type of estimate	Upper limit	Lower limit	Point estimate	No. of countries	No. of studies or data sets^a^	Frequency type	Population source	Denominator	Assessment/diagnostic method
Pregnancy												
Unsafe severe abortion complications (ratio)	Adler, 2012^54^	LMICs	Median	5.30%	0.435%	0.60%	8	9	Incidence	Population	Live births	Clinical review
Placenta previa	Cresswell, 2013^44^	World	Weighted mean	0.59%	0.45%	0.52%	25	41	Prevalence	Population^b^	Deliveries and live births	Clinical confirmation, not reported for 17/41 studies
Nausea and vomiting	Einarson, 2013^23^	World	Weighted average rate	72.30%	66.50%	69.40%	13	59	Prevalence	Not clear	Pregnancies	Not clear
Hyperemesis gravidarum	Einarson, 2013^23^	HICs	Weighted average rate	3.60%	0.20%	1.20%	8	18	Prevalence	Not clear	Pregnancies	Not clear
Gestational diabetes	Buckley, 2012^13^	HICs	Range	22.30%	0.70%		20	33	Prevalence	Population	Pregnancies, deliveries	Several diagnostic criteria, 1 study with unknown criteria
Gestational diabetes	Hirst, 2012^14^	Asia	Range	17.70%	0.56%		7	19	Prevalence	Population^b^	Pregnancies	Diagnostic criteria included WHO, Japan Society of Obstetrics and Gynecology, NDDG, O'Sullivan and Mahan,^71^ and Carpenter and Coustan^72^
Gestational diabetes	Hunt, 2007^15^	World	Range	22.30%	1.20%		16	26	Prevalence	Population	Live births, pregnancies, unclear	Information missing for several studies. Included 1 study using self‐report
Gestational diabetes	Kanguru, 2014^19^	LMICs	Range	17.25%	0.40%		8	12	Prevalence	Population	Pregnancies	Diagnostic criteria included WHO, ADA and IADSPSG
Gestational diabetes	Macaulay, 2014^16^	Africa	Range	13.90%	0%		6	14	Prevalence	Not clear	Not clear	Several diagnostic criteria, including institutional protocols based on fast blood glucose
Gestational diabetes	Mwanri, 2015^17^	Africa	Weighted mean	10.01%	1.68%	5.06%	5^c^	13^c^	Prevalence	Not clear	Not clear	Not clear
Gestational diabetes	Schneider, 2012^18^	HICs	Range	11.60%	1.70%		10	27	Prevalence	Population	Pregnancies, births	Self‐report/insulin test/glucose therapy/clinical diagnosis
Gestational diabetes	Zhu, 2016^20^	Europe	Median	22.30%	1.80%	5.80%	12	Not clear	Prevalence	Not clear	Not clear	Several diagnostic criteria, some of which were not specified
Gestational diabetes	Zhu, 2016^20^	North America and Caribbean	Median	11.90%	6.50%	7.00%	4	Not clear	Prevalence	Not clear	Not clear	Several diagnostic criteria, some of which were not specified
Gestational diabetes	Zhu, 2016^20^	South and Central America	Median	16.60%	7.10%	11.20%	2	Not clear	Prevalence	Not clear	Not clear	Several diagnostic criteria, some of which were not specified
Gestational diabetes	Zhu, 2016^20^	Middle East and North Africa	Median	24.50%	8.40%	12.90%	5	Not clear	Prevalence	Not clear	Not clear	Diagnostic criteria included WHO, NDDG, IADSPG, Carpenter and Coustan^72^
Gestational diabetes	Zhu, 2016^20^	Africa	Median	9.50%	8.20%	8.90%	2	Not clear	Prevalence	Not clear	Not clear	Diagnostic criteria included WHO and IADPSG
Gestational diabetes	Zhu, 2016^20^	South East Asia	Median	18.30%	8.10%	11.70%	4	Not clear	Prevalence	Not clear	Not clear	Several diagnostic criteria, some of which were not specified
Gestational diabetes	Zhu, 2016^20^	Western Pacific	Median	25.10%	4.50%	11.70%	7	Not clear	Prevalence	Not clear	Not clear	Several diagnostic criteria, some of which were not specified
Around delivery												
Retained placenta (3rd stage of labor, >30 min)	Cheung, 2011^55^	HICs	Median	6.26%	2.00%	2.67%	4	6	Incidence	Not clear	Vaginal deliveries	3rd stage labor at >30 min, but not clear how recorded or by whom
Retained placenta (3rd stage of labor, >30 min)	Cheung, 2011^55^	LMICs	Median	4.60%	1.05%	1.55%	3	3	Incidence	Not clear	Vaginal deliveries	3rd stage labor at >30 min, but not clear how recorded or by whom
Retained placenta (MROP)	Cheung, 2011^55^	HICs	Median	5.42%	0.60%	2.40%	6	9	Incidence	Not clear	Vaginal deliveries	MROP, but not clear how recorded or by whom
Retained placenta (MROP)	Cheung, 2011^55^	LMICs	Median	0.57%	0.008%	0.43%	4	6	Incidence	Not clear	Vaginal deliveries	MROP, but not clear how recorded or by whom.
Pre‐eclampsia	Abalos, 2014^31^	World	Mean (range)	4.20%	1.20%	2.30%	31	52^a^	Incidence	Both	Deliveries	Not clear: For 17% of pre‐eclampsia studies the outcome definition was not clear
Eclampsia	Abalos, 2014^31^	World	Mean (range)	2.70%	0.10%	1.10%	28	42^a^	Incidence	Both	Deliveries	Not clear: For 45% of eclampsia studies the outcome definition was not clear
Postpartum hemorrhage (≥500 mL)	Calvert, 2012^29^	World	Weighted mean	12.10%	9.60%	10.80%	29	63	Prevalence	Population^b^	Deliveries	Objective, subjective, and unknown (21/104 data sets) methods of blood loss measurement were included
Postpartum hemorrhage (≥500 mL)	Carroli, 2008^30^	World	Weighted mean	6.05%	6%	6.02%	Not clear	14	Prevalence	Population	Deliveries	Objective, subjective, and unspecified (14/55 studies) methods of blood loss measurement were included
Severe postpartum hemorrhage (≥1000 mL)	Calvert, 2012^29^	World	Weighted mean	3.20%	2.40%	2.80%	27	37	Prevalence	Population^b^	Deliveries	Objective, subjective, and unknown (6/69 data sets) methods of blood loss measurement were included
Severe postpartum hemorrhage (≥1000 mL)	Carroli, 2008^30^	World	Weighted mean	1.71%	1.64%	1.67%	Not clear	4	Prevalence	Population	Deliveries	Objective, subjective, and unspecified (2/25 studies) methods of blood loss measurement were included
Amniotic fluid embolism	Conde‐Agudelo, 2009^25^	North America	Weighted mean	0.0072%	0.0060%	0.0066%	2	3	Incidence	Population	Deliveries	Not clear
Amniotic fluid embolism	Conde‐Agudelo, 2009^25^	Europe	Weighted mean	0.0021%	0.0017%	0.0019%	3	3	Incidence	Population	Deliveries	Not clear
Amniotic fluid embolism	Frati, 2014^56^	Not clear	Mean (range)	0.02%	0.00%	0.01%	Not clear	8	Incidence	Not clear	Deliveries	Clinical assessment
3rd‐ and 4th‐degree perineal tear	Villot, 2015^24^	Not clear	Range	9.70%	2.95%	6.3250%	Not clear	3	Prevalence	Not clear	Not clear	Not clear
Pregnancy and postpartum												
Deep vein thrombosis	Kourlaba, 2016^26^	World	Weighted mean	0.11%	0.10%	0.11%	7^c^	9^c^	Incidence	Not clear	Pregnant and postpartum women	Not clear
Deep vein thrombosis	Meng, 2015^57^	World	Weighted mean	1.30%	1.00%	1.10%	10	18	Incidence	Not clear	Pregnant and postpartum women	Clinical review or tests (e.g. ultrasound).

Abbreviations: ADA, American Diabetes Association; EPDS, Edinburgh Postnatal Depression Scale; EASD, European Foundation for the Study of Diabetes; HICs, high‐income countries; IADSPSG, International Association of the Diabetes and Pregnancy Study Groups; LMICs, low‐ and middle‐income countries; NDDG, National Diabetes Data Group; MROP, manual removal of placenta.

a Number of data sets.

b Only included hospital‐based studies if the region in which the study was conducted had at least 95% of births attended by a skilled birth attendant. For Hirst et al.,^14^ this is because LMICs included have universal screening.

c It was not clear whether the details for this matched the frequency estimate extracted.

**Table 2:** Indirect maternal morbidity estimates

Condition	Author	Group of countries	Type of estimate	Upper limit	Lower limit	Point estimate	No. of countries	No. of studies or data sets^a^	Prevalence vs incidence	Population source	Denominator	Assessment method
Pregnancy												
Pre‐existing diabetes mellitus	Kanguru, 2014^19^	LMICs	Range	0.70%	0.00%		6	7	Prevalence	Both	Pregnancies	Diagnostic criteria included WHO, NDDG, EASD
Malaria (peripheral parasitemia)	Chico, 2012^27^	Eastern and Southern Africa	Weighted mean	36.50%	22.40%	29.50%	8	19	Prevalence	Facility (ANC)	Women attending ANC	Laboratory
Malaria (peripheral parasitemia)	Chico, 2012^27^	West and Central Africa	Weighted mean	41.90%	28.20%	35.10%	8	36	Prevalence	Facility (ANC)	Women attending ANC	Laboratory
Malaria (placental parasitemia)	Chico, 2012^27^	Eastern and Southern Africa	Weighted mean	36.40%	16.70%	26.50%	5	9	Prevalence	Facility (ANC)	Women attending ANC	Laboratory
Malaria (placental parasitemia)	Chico, 2012^27^	West and Central Africa	Weighted mean	47.60%	28.40%	38.00%	6	15	Prevalence	Facility (ANC)	Women attending ANC	Laboratory
Hepatitis B (seroprevalence of HBsAg)	Merrill, 2011^58^	World	Median			4.30%	52^b^	98^b^	Prevalence	Both	Pregnancies	Laboratory
HIV	Drake, 2014^36^	Africa	Weighted mean	6.10%	3.30%	4.70%	13	16	Incidence	Not clear	Pregnancies (person years at risk)	Laboratory
Hepatitis C	Mora, 2016^37^	Sub‐Saharan Africa	Weighted mean	4.28%	1.46%	2.51%	Not clear	18	Prevalence	Not clear	Not clear	Laboratory
Hepatitis C	Rao, 2015^38^	Sub‐Saharan Africa	Random effects model	3.84%	2.23%	3.04%	10	21	Prevalence	Facility (ANC)	Pregnancies	Laboratory
Hepatitis C	Riou, 2016^59^	LMICs	Range	9.20%	0.20%		15	28	Prevalence	Not clear	Pregnancies	Laboratory
Chlamydia	Chico, 2012^27^	Eastern and Southern Africa	Weighted mean	7.10%	3.40%	5.20%	6	5	Prevalence	Facility (ANC)	Women attending ANC	Laboratory
Chlamydia	Chico, 2012^27^	West and Central Africa	Weighted mean	3.50%	0.20%	1.90%	2	2	Prevalence	Facility (ANC)	Women attending ANC	Laboratory
Chlamydia	Joseph Davey, 2016^60^	Eastern Africa	Adjusted mean	5.60%	2.80%	4.20%	6^b^	3	Prevalence	Both: estimates adjusted by setting	Pregnancies	Laboratory
Chlamydia	Joseph Davey, 2016^60^	Southern Africa	Adjusted mean	6.60%	2.30%	4.40%	6^b^	3	Prevalence	Both: estimates adjusted by setting	Pregnancies	Laboratory
Chlamydia	Joseph Davey, 2016^60^	Latin America	Adjusted mean	16.40%	6.00%	11.20%	5^b^	7	Prevalence	Both: estimates adjusted by setting	Pregnancies	Laboratory
Chlamydia	Joseph Davey, 2016^60^	Asia	Adjusted mean	1.10%	0.40%	0.80%	9^b^	6	Prevalence	Both: estimates adjusted by setting	Pregnancies	Laboratory
Syphilis	Chico, 2012^27^	Eastern and Southern Africa	Weighted mean	3.60%	2.10%	2.90%	8	17	Prevalence	Facility (ANC)	Women attending ANC	Laboratory
Syphilis	Chico, 2012^27^	West and Central Africa	Weighted mean	4.60%	0.40%	2.50%	4	5	Prevalence	Facility (ANC)	Women attending ANC	Laboratory
Syphilis	Joseph Davey, 2016^60^	Eastern Africa	Adjusted mean	5.40%	3.70%	4.60%	6	8	Prevalence	Both: estimates adjusted by setting	Pregnancies	Laboratory
Syphilis	Joseph Davey, 2016^60^	West Africa	Adjusted mean	6.30%	1.70%	4.00%	4^b^	4	Prevalence	Both: estimates adjusted by setting	Pregnancies	Laboratory
Syphilis	Joseph Davey, 2016^60^	Southern Africa	Adjusted mean	8.30%	4.70%	6.50%	6^b^	8	Prevalence	Both: estimates adjusted by setting	Pregnancies	Laboratory
Syphilis	Joseph Davey, 2016^60^	Latin America	Adjusted mean	3.30%	1.20%	2.20%	5^b^	15	Prevalence	Both: estimates adjusted by setting	Pregnancies	Laboratory
Syphilis	Joseph Davey, 2016^60^	Asia	Adjusted mean	1.60%	0.50%	1.10%	9^b^	13	Prevalence	Both: estimates adjusted by setting	Pregnancies	Laboratory
Anogenital warts	Banura, 2013^61^	Africa	Range	7.30%	0.20%		5	11	Prevalence	Both	Pregnancies	Clinical review
Intimate partner violence	Liepe, 2013^62^	HICs	Range	31.70%	1.80%		7	12	Prevalence	Not clear	Pregnancies	All validated questionnaires
Intimate partner violence	Puccia, 2012^63^	World	Range	94%	3.40%		9	16	Prevalence	Not clear	Pregnancies	5 studies not reported, 1 self‐reported, remaining used validated scales
Intimate partner violence	Shamu, 2011^64^	Africa	Weighted mean	16.08%	14.38%	15.23%	4	13	Prevalence	Both	Pregnancies	Mixture of “own” tool with validated scales
Depression	Sawyer, 2010^35^	Africa	Weighted mean	9.50%	13.10%	11.30%	3	5	Prevalence	Not clear	Pregnancies	20 studies conducted clinical interviews, 10 used self‐administered measures and 3 used both
Depression (moderate to severe)	Schmied, 2013^65^	HICs	Range	20.50%	8.70%		2	2	Prevalence	Not clear	Pregnancies	A variety of scales was used to assess depression; EPDS was the most often used
Any anxiety disorder	Goodman, 2014^28^	World	Range	39.00%	4.40%		8	10	Prevalence	Facility (ANC)	Pregnancies	Validated scale or clinical interview
Anxiety	Sawyer, 2010^35^	Africa	Weighted mean	12.30%	17.40%	14.80%	1	2	Prevalence	Not clear	Pregnancies	Validated scale or clinical interview
Bipolar disorder	Sharma, 2012^66^	HICs	Range	1.40%	0%		4	4	Prevalence	Population	Pregnancies	Interviews and self‐reported scales
Generalized anxiety disorder	Goodman, 2014^28^	World	Range	10.50%	0.00%		9	11	Prevalence	Facility (ANC)	Pregnancies	Validated scale or clinical interview
Panic disorder	Goodman, 2014^28^	World	Range	5.70%	0.20%		9	12	Prevalence	Facility (ANC)	Pregnancies	Validated scale or clinical interview
Post‐traumatic stress disorder	Goodman, 2014^28^	World	Range	7.90%	0.00%		8	13	Prevalence	Facility (ANC)	Pregnancies	Validated scale or clinical interview
Carpal tunnel syndrome	Padua, 2010^67^	Unclear region	Range	43.00%	7%		Not clear	5	Prevalence	Population	Pregnancies	Neurophysiologically confirmed
Urinary incontinence	Cerruto, 2013^4^	HICs	Range	58.10%	6.70%		Not clear	6	Prevalence	Population	Pregnancies	Questionnaires, some validated; 1 study no clear information
Urinary incontinence	Sangsawang, 2013^39^	HICs	Range	75.00%	26%		4	5	Prevalence	Population	Pregnancies	Not clear
Postpartum												
HIV	Drake, 2014^36^	Africa	Weighted mean	4.00%	1.80%	2.90%	3	7	Incidence	Not clear	Postpartum women (person years at risk)	Laboratory
Depression (minor and major)	Norhayati, 2015^33^	HICs	Range	62.00%	0.10%		11	16	Prevalence	Not clear	Postpartum women	Clinical interviews
Depression (minor and major)	Norhayati, 2015^33^	LMICs	Range	26.30%	1%		4	5	Prevalence	Not clear	Postpartum women	Clinical interviews
Major depressive disorders	Norhayati, 2015^33^	World	Range	62.00%	0.10%		15	21	Prevalence	Not clear	Postpartum women	Clinical interviews
Depression	Parsons, 2012^34^	LMICs	Range	50%	4.90%		28	84	Prevalence	Not clear	Postpartum women	Validated scales or clinical interviews
Depression	Sawyer, 2010^35^	Africa	Weighted mean	19.10%	17.60%	18.30%	6	21	Prevalence	Not clear	Postpartum women	Validated scales or clinical interviews
Depression (moderate to severe)	Schmied, 2013^65^	HICs	Range	16.00%	9.00%		2	Not clear	Prevalence	Not clear	Postpartum women	Validated scales or clinical interviews
Post‐traumatic stress disorder	Goodman, 2016^68^	World	Weighted mean	4.58%	0.66%	1.78%	4	6	Prevalence	Both	Postpartum women	Validated scales or clinical interviews
Post‐traumatic stress disorder	Grekin, 2014^69^	World	Weighted mean	3.90%	2.50%	3.10%	13	41	Prevalence	Population	Postpartum women	Validated scales or clinical interviews
Panic disorder	Goodman, 2016^68^	World	Weighted mean	2.76%	0.09%	1.66%	4	6	Prevalence	Both	Postpartum women	Validated scales or clinical interviews
Anxiety disorder not otherwise specified (NOS)	Goodman, 2016^68^	HICs	Weighted mean	4.91%	0.01%	0.38%	2	2	Prevalence	Population	Postpartum women	Clinical interviews
Any anxiety disorder	Goodman, 2016^68^	World	Weighted mean	13.83%	5.17%	8.56%	5	6	Prevalence	Population	Postpartum women	Validated scales or clinical interviews
Generalized anxiety disorder	Goodman, 2016^68^	World	Weighted mean	6.66%	1.85%	3.59%	5	8	Prevalence	Both	Postpartum women	Validated scales or clinical interviews
Anxiety	Sawyer, 2010^35^	Africa	Weighted mean	15.20%	12.90%	14.00%	2	2	Prevalence	Not clear	Postpartum women	Validated scales or clinical interviews
Urinary incontinence	Cerruto, 2013^4^	HICs	Range	31.00%	3.00%		Not clear	6	Prevalence	Population	Postpartum women	Questionnaires, some validated; 1 study no clear information
Urinary incontinence	Thom, 2010^42^	HICs	Mean	36.00%	32.00%	33.00%	9	5	Prevalence	Population^c^	Postpartum women at 3 months	Not clear
Obstetric fistula	Adler, 2013^32^	LMICs	Weighted mean	0.11%	0%	0.03%	9	10	Prevalence	Population	Women of reproductive age	Physical exam
Obstetric fistula	Cowgill, 2015^40^	LMICs	Range	0.41%	0.03%		9	4	Prevalence	Population	Deliveries	Physical exam (not clear for modeled estimate from Nigeria)
Obstetric fistula	Zheng, 2009^41^	LMICs	Range	1.56%	0.01%		7	2	Incidence	Population	Deliveries/live births	Unvalidated questionnaires and physical exam
Pregnancy and postpartum												
Malaria	Roberts, 2011^70^	LMICs	Range	78.69%	0%		13	43	Prevalence	Both	Pregnant and postpartum women up to 42 days	Unclear, no information on 21% of the tests, remaining were laboratory‐based
Pulmonary embolism	Kourlaba, 2016^26^	World	Weighted mean	0.06%	0.02%	0.04%	7^b^	7^b^	Incidence	Not clear	Deliveries, pregnant and postpartum women	Not clear
Pulmonary embolism	Meng, 2015^57^	World	Weighted mean	0.04%	0.02%	0.03%	10	18	Incidence	Not clear	Deliveries	Clinical review and diagnostic tests (e.g. ultrasound)

Abbreviations: ADA, American Diabetes Association; EPDS, Edinburgh Postnatal Depression Scale; EASD, European Foundation for the Study of Diabetes; HICs, high‐income countries; IADSPSG, International Association of the Diabetes and Pregnancy Study Groups; LMICs, low‐ and middle‐income countries; NDDG, National Diabetes Data Group; ANC, antenatal care.

a Number of data sets.

b It was not clear whether the details for this matched the frequency estimate extracted.

c They define inclusion criteria for population as “studies on incontinence in population‐based sample defined as from one or more district hospitals or from multiple clinics covering a defined geographic area.” However, two countries contributing to the estimates were Turkey and Iran, for which hospital recruitment might not always be entirely appropriate.

The systematic reviews covered different geographical and economic areas, e.g. the world, high‐income countries (HICs), low‐ and middle‐income countries (LMICs), or specific regions (Africa, Asia, Europe, etc.). Of the estimates we extracted, 17 (18%) were based on only two countries or it was not clear from the paper how many countries were included. Among the frequency estimates that included worldwide studies, the median number of countries contributing data was 10 (interquartile range, 7.5–20.5). Sub‐Saharan Africa was the world region with the highest number of specifically dedicated systematic reviews (n=9).

Tables 1 and 2 describe the outcome assessment method behind each estimate. Information on assessment method at the estimate level was often scarce and poorly described. For direct morbidity estimates, the information on the assessment method underlying the estimates was unclear in five systematic reviews.^17^, ^23^, ^24^, ^25^, ^26^ In addition, some studies used assessment methods that are prone to bias. To take the example of gestational diabetes, Schneider et al.,^18^ and Hunt and Schuller 15 included primary studies that used self‐report. Zhu and Zhang,^20^ on the other hand, reported clearly on diagnostic criteria at the estimate level.

### 3.3 Quality of systematic reviews

**Figure 2:**
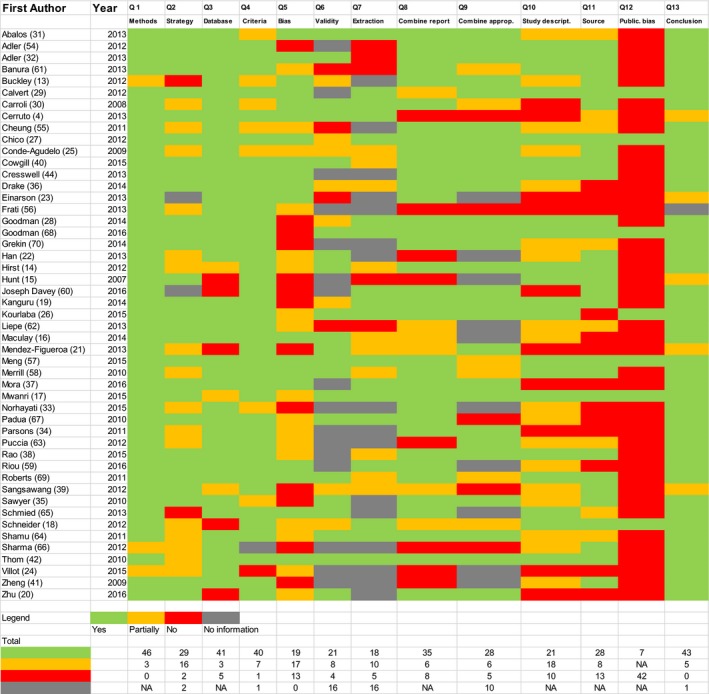
Quality assessment.

There was much variation in the quality of the 49 systematic reviews, including some examples of excellent methodology and reporting.^27^, ^28^, ^29^ Some aspects of quality were often found to be particularly poor, including insufficient reporting and methodological gaps (Fig. 2). For example, only 19 (39%) of the systematic reviews explicitly reported their language exclusions and the inclusion of grey literature, and only 21 (43%) provided a detailed description of the primary studies. Furthermore, for 16 (33%) of the reviews we did not have sufficient details on the data extraction process (e.g. use of independent extraction).

Information on data collection and sources was also lacking in many cases: for 19 (56%) of the direct morbidity estimates and 18 (30%) of the indirect estimates there was insufficient information to assess whether the data were from population‐ or facility‐based sources. Overall, 32 (34%) of the estimates extracted included data from facility‐based studies. Facility‐based studies vary in terms of their representativeness. For example, when reviewing studies of the prevalence of malaria, Chico et al.^27^ included women attending antenatal care clinics, a service that currently most African women attend at least once during pregnancy. Nevertheless, as the authors indicate, these estimates are only representative of those who attended antenatal care, and this paper includes studies from Africa going back at least two decades when antenatal care attendance was much lower.

One‐third (n=16; 32%) of the systematic reviews did not explicitly report whether they performed a quality assessment of their primary studies. Even when a quality assessment was conducted, most studies did not use a standardized tool or did not report which tool they used or the results. Publication bias was assessed by only 15% of the systematic reviews.

### 3.4 Frequency of maternal morbidity along the pregnancy–postpartum continuum

**Figure 3:**
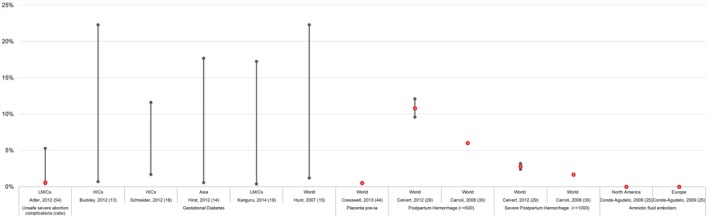
Population‐based estimates for direct maternal morbidities. Abbreviations: LMICs, low‐ and middle‐income coutries; HICs, high‐income countries. [Colour figure can be viewed at http://www.wileyonlinelibrary.com]

As shown in Table 1 and Figure 3, the toll of potentially life‐threatening direct maternal morbidities is high, with postpartum hemorrhage being the most common, estimated at 6.2% based on the review by Carroli et al.,^30^ and at 10.8% based on the more recent review with different population‐based criteria by Calvert et al.^29^ This is followed by pre‐eclampsia (2.3%),^31^ severe abortion complications (0.6%),^32^ and eclampsia (0.5%)^31^ (Table 1). Substantial direct maternal morbidity is also present throughout pregnancy with the prevalence of gestational diabetes mellitus estimated to be 5.1% in Africa^17^ and 25.1% in the Western Pacific Region (Table 1, Fig. 1).^20^

**Figure 4:**
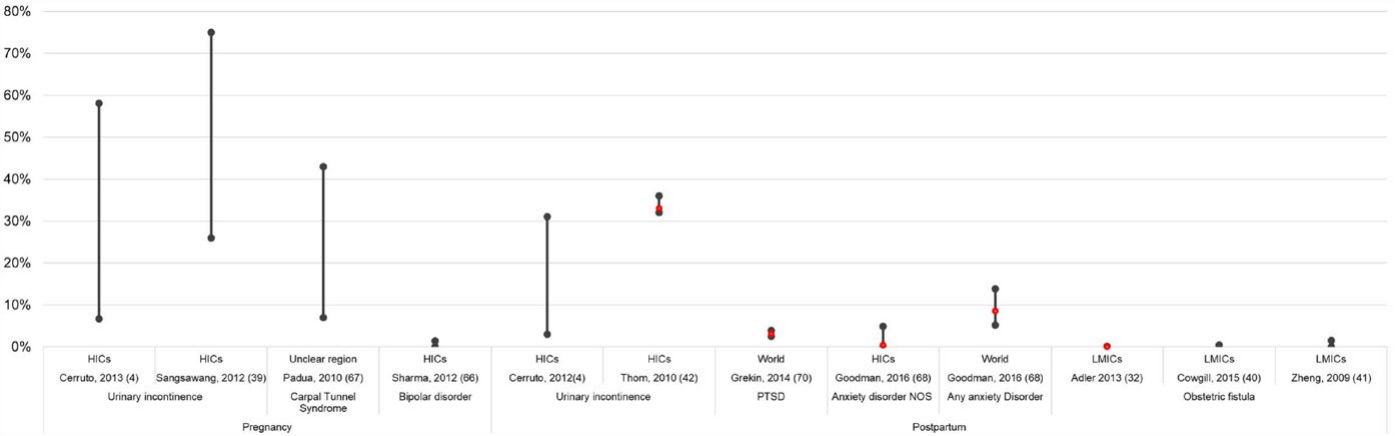
Population‐based estimates for indirect maternal morbidities. Abbreviations: LMICs, low‐ and middle‐income countries; HICs, high‐income countries. [Colour figure can be viewed at http://www.wileyonlinelibrary.com]

The frequency of indirect maternal morbidity is also high (see Table 2 and Fig. 4), particularly for mental health and infectious diseases. The prevalence of postpartum depression estimated for LMICs ranged from 1.0% to 26.3% according to Norhayati et al.^33^ and from 4.9% to 50% according to Parsons et al.^34^ In Africa, Sawyer et al.^35^ estimated the prevalence of pregnancy‐related depression at 18.3%. Anxiety is another common health problem, with prevalence worldwide ranging between 4.4% and 39.0%^28^ during pregnancy, and estimated to affect 8.5% of postpartum women on average.^28^ The average prevalence of anxiety during pregnancy and the postpartum period in Africa has been estimated at 14%^35^ (Table 2).

Regarding infectious diseases,^36^ the estimated pooled HIV incidence rate in Sub‐Saharan Africa is 4.7 per 100 person‐years during pregnancy and 2.9 per 100 person‐years during the postpartum period (Table 2). In Sub‐Saharan Africa, based on one systematic review,^27^ the reported prevalence of syphilis and chlamydia during pregnancy ranged between 2.5% and 2.9% and between 1.9% and 5.2%, respectively. Estimates of these conditions across LMICs, as reported in another systematic review, range between 0.5% and 8.3% for syphilis and between 0.4% and 16.4% for chlamydia (Table 2). Across Sub‐Saharan Africa, prevalence of malaria during pregnancy (peripheral parasitemia) ranges between 29.5% (in Eastern and Southern Africa) and 35.1% (in Western and Central Africa).^27^ Estimates for hepatitis are high, with a median of 4.3% of pregnancies diagnosed with seroprevalence of hepatitis B serum antigen (HBsAg), and between 2.5%^37^ and 3.0%^38^ of pregnant women in Africa infected with hepatitis C (Table 2).

Many pregnancies are affected by non‐life‐threatening conditions. Based on evidence predominately from HICs, nausea and vomiting have been reported to affect 69.4% of pregnant women^23^ (Table 1). Similarly, based on data from HICs, urinary incontinence has been variously estimated to affect 6.7% to 58.1%^4^ or 26.0% to 75.0%^39^ of women during pregnancy (Table 2).

Obstetric fistula, experienced by under 1% of postpartum women in LMICs, is one of the more severe although less prevalent maternal morbidities.^32^, ^40^, ^41^ Unrepaired fistulae can impact a woman's health and well‐being severely for the rest of her lifetime. Similarly, postpartum urinary incontinence can persist for a lifetime, and currently affects, on average, 33.0% of women during the puerperium in HICs according to one review,^42^ or between 3.0% and 31.0% as estimated in another review.^4^

For some conditions, such as mental health disorders and infections, the timing of diagnosis may influence the frequency of the condition and thus explain differences in estimates between studies; this detail was not always reported. In Appendix S5, we summarize the case for postpartum depression, for which there are notable differences between the systematic reviews in how they summarized data from longitudinal studies reporting prevalence data for more than one time point.

## 4 DISCUSSION

We conducted a systematic review of systematic reviews assessing the frequency of the 121 WHO maternal morbidities.^5^ Women suffer substantial morbidity during pregnancy, at the time of birth, and in the postpartum period. This review also identified important knowledge gaps. Surprisingly, no systematic reviews were available for several maternal conditions known to be potentially life‐threatening and some that can lead to long‐term disabilities, such as puerperal infection and anemia. Another important finding is that the quality of many of the 49 included systematic reviews was poor. Key areas for improvement include the strict inclusion of population‐based (rather than facility‐based) studies and improvement in the reporting of methods in line with available guidelines.

The importance of the time around delivery was traditionally emphasized in maternal health research as most maternal deaths occur in this period. However, our results show that the burden of maternal morbidity is also high before and after the point of delivery. Gestational diabetes affects at least 5% of women in low‐resource settings. Poor mental health is also common during pregnancy and in the postpartum period, with depression and anxiety the most common conditions. Infectious diseases are also frequent, including HIV, malaria, and hepatitis. These data call into question the completeness of currently available estimates on the overall burden of maternal morbidity (such as the ones provided by the GBD study group), as they rely on a limited number of highly prevalent maternal conditions, excluding, for example, gestational diabetes and anxiety.

Nevertheless, we have limited ability to comment on the frequency of 71% of the conditions listed by Chou et al.^5^ owing to the lack of systematic reviews for them. Furthermore, systematic reviews aiming to provide global estimates for a condition included a median of only 10 countries, which also casts some doubt on the geographical representativeness of currently available estimates of the burden of disease. This state of affairs has a number of possible explanations: (1) that maternal morbidity is not a research priority; (2) that some conditions are challenging to define and measure; and (3) that others are very rare and hence unlikely to be covered in a systematic review. Information on a wider range of maternal conditions and geographical areas should be gathered to produce better estimates.

The differences in prevalence reported for the same conditions, such as gestational diabetes and depression, may reflect actual differences between the populations and the widening inequalities between and within regions,^2^ but they are also likely to be driven by methodological differences between the systematic reviews and the primary studies they included. Potential drivers include different assessment methods, varying definitions of the condition, and differences in the study populations and the timing of assessment. For example, the type of assessment method applied can double prevalence estimates for a condition such as gestational diabetes.^16^ Generally, however, estimates for the same condition were relatively consistent; for example, estimates for postpartum hemorrhage varied between 6% and 11%, and obstetric fistula between 0% and 1.6% in LMICs.

Our results also highlight the existing gaps in the quality of methods and reporting used in systematic reviews on maternal conditions. Crucially, for 56% of the direct and 30% of the indirect estimates, there was insufficient information to verify the population or data source. Overall, 34% of the estimates extracted included facility‐based studies. As discussed elsewhere, more reliable population‐based estimates are needed, since mothers who access facilities are likely to be different to the ones who do not.^43^ Lack of facility attendance by women during pregnancy, delivery, and the postpartum period could lead to underestimation of the frequency of some conditions (e.g. if women tend not to seek help for that condition) or overestimation (e.g. if women with serious morbidity are more likely to attend a facility). Few systematic reviews used a rigorous method to select available data from LMICs for inclusion, such as only including hospital‐based studies if the region in which the study was conducted had at least 95% of births attended by a skilled birth attendant.^29^, ^44^ Other important limitations among the included systematic reviews included the potential for study selection bias, the inadequate use of quality‐assessment tools, reporting insufficient detail on the data extraction process, a poor description of the primary studies included, and lack of clarity about the diagnostic tools used to generate the estimates provided. Our findings on the poor quality of systematic reviews resonates with those of Sheick et al.,^45^ who reviewed the quality of systematic reviews on maternal medicine in 2007. A decade on, there is still much room for improvement.

We propose key steps to improve the quality of systematic reviews in the context of maternal morbidity, including the quality of the methods used to conduct them and the quality of the reporting. Our recommendations are similar to those proposed for estimating newborn morbidity^46^ and health estimates more broadly.^47^ These recommendations are addressed to the authors of systematic reviews, primary studies, study reviewers, and journal editors. First and foremost, researchers should use and report on studies according to standard guidelines for the review of observational studies, such as the PRISMA and STROBE guidelines.^6^, ^48^, ^49^, ^50^ In particular, we encourage the reporting of details on the eligible primary studies, including data source, sample size, and country.

Other important recommendations include:

1. Explicitly report the data sources (facility‐ and/or population‐based) used to generate frequency estimates and for each primary study included. The gold standard is to restrict inclusion to primary studies that are population‐based, or restrict to those studies from geographical areas where the majority of women attend facility‐based services if included studies use facility‐based recruitment. If this is not possible, pooled estimates should be reported separately for studies that used population‐based and facility‐based data collection.
2. Specify what assessment methods were used for each overall estimate presented. It is also good to report different summary estimates by diagnostic criteria. Try and avoid studies that include self‐reported data except when this is an acceptable way of measuring the condition (e.g. nausea and vomiting). If self‐reporting is included, discuss the primary studies assessing the validity of the self‐report (sensitivity and specificity).
3. State the denominator used. Preferably prioritize pregnancies and postpartum women with clear definitions of this period (e.g. length of time postpartum, etc.).
4. Use appropriate and standardized regional classifications based on the final list of primary studies included in the summary estimates provided.
5. Provide frequency estimates at different points of the pregnancy–postpartum continuum, if relevant to the condition of interest.

Whether conditions arising during pregnancy should be quantified as incidence or prevalence heavily depends on the condition of interest, and the design and aims of a study. Yet many researchers use these terms interchangeably in the context of maternal morbidity; this is an issue that is beyond the scope of this study. However, we found that reviews of certain conditions for which incidence is of interest, such as postpartum depression, reported solely on prevalence. In systematic reviews, where several primary studies with a variety of designs are included, it can be difficult to choose the type of frequency to report. We call for future systematic reviews to clearly distinguish between incidence and prevalence estimates, to disaggregate these data, and to provide more discussion on this issue.

Our systematic review of systematic reviews is limited by the lack of grading based on diagnostic criteria. We chose not to perform such assessment because the primary studies in the included systematic reviews spanned across several conditions and decades, during which time the appropriateness of diagnostic criteria for different conditions changed. A further limitation is that we did not extract information directly from the primary studies identified by the systematic reviews—some systematic reviews included the same primary studies, and we did not always limit the time period for the publication of these primary studies—hence our reported frequencies represent a wide timescale. Overall, our review is limited by the quality of both the included systematic reviews and the primary studies they covered.

Finally, we only searched for systematic reviews rather than primary studies to assess the frequency of these conditions. We are aware of large‐scale analyses of the frequency of important conditions such as anemia,^51^ pregnancy‐related infection,^52^ and fistula,^53^ which provide robust estimates for these conditions. We chose to focus, however, on systematic reviews that use standardized methods to aggregate existing data.

In conclusion, this review highlights both the existence of substantial maternal morbidity—spanning the time before and beyond childbirth—and major remaining gaps in the availability of systematic reviews for some maternal morbidities. Future systematic reviews should improve their quality standards, including the strict inclusion of population‐based studies, and improvement of their review methods and their reporting, following available guidelines. With the changing burden of poor maternal health across the globe related to the obstetric transition, there is a pressing need to strengthen the evidence base for prioritizing action and further research. A central repository where results from new systematic reviews, using standardized terminology and metrics, can be stored and readily shared would be invaluable in tracking this shifting burden and in informing interventions to reduce the impact of maternal morbidities on women's lives.

## AUTHOR CONTRIBUTIONS

GG, WJG, SW, and VF designed the research questions and methods. AL, GG, CC, and SW conducted data extraction and analysis. GG prepared the manuscript. All authors (GG, AL, CC, WJG, SW, VF) provided feedback on the manuscript.

## CONFLICTS OF INTEREST

The authors have no conflicts of interest to declare.

## Supporting information


**Appendix S1.** Search strategy.

**Appendix S2.** Quality assessment methods.

**Appendix S3.** List of available systematic reviews.

**Appendix S4.** Details of direct and indirect morbidity estimates.

**Appendix S5.** Timing of postpartum depression assessment.
