# Supplementary material for: The frequency of maternal morbidity: A systematic review of systematic reviews
Source: Int J Gynaecol Obstet. 2018 May 23;141(Suppl Suppl 1):20–38. doi: 10.1002/ijgo.12468 (PMC6001670; doi:10.1002/ijgo.12468)
Supplement: Supplementary file 1 — Appendix S1. Search strategy. [file IJGO-141-20-s001.docx]

**Appendix S1. Search strategy**

**Objective**: Identify all systematic reviews available that capture the magnitude of maternal morbidities (both mild and severe conditions), worldwide and/or for major world regions from 2005.

Carry out a systematic review of systematic reviews that comply with the following preliminary criteria:

**Search strategy**

1. Maternal text
2. Maternal MeSH
3. 1 OR 2
4. Frequency text
5. Frequency MeSH
6. 4 OR 5
7. Type of publications text
8. Type of publications MeSH
9. 7 OR 8
10. Individual morbidity terms e.g. (hypertensive disorders OR ectopic pregnancy OR etc.)
11. Pregnancy complications MeSH
12. 10 OR 11
13. 3 AND 6 AND 9 AND 12
14. Unsafe abortion terms
15. 6 AND 9 AND 14
16. 13 OR 15
17. Restricted to humans

**Inclusion criteria**

1. The condition is in the WHO maternal morbidity list.
2. Provides frequency of outcome for maternal conditions of interest.
3. The most recently published of the included studies was published after 2006 (with the aim of having recent data to update the evidence since the last *Lancet* maternal health series, which was published in 2006).
4. Provide world or regional estimates (at least two countries).

**Exclusion criteria**

1. The review is restricted to a certain subgroup e.g. rural women, or women with a specific health condition, twins, women with a previous cesarean delivery.
2. The publication was a case report or nonsystematic review.
3. Studies focused on risk factors or consequences of a certain maternal condition.
4. Studies primarily included interventions or investigated the effect of a single individual characteristic of the relevant maternal conditions.
5. Does not mention frequency of the outcome among pregnant women or special groups in the abstract.

*Extraction table – Table 1 and Appendix S4.*

*Quality appraisal – Appendix S2.*

*Pregnancy terms:*

| *Text* | *Embase* | *MEDLINE* | *CINAHL* |
| --- | --- | --- | --- |
| matern* OR pregnan* OR antepartum OR antenatal OR pre?natal OR perinatal OR childbirth OR intrapartum OR intra-partum OR postpartum OR post-partum OR puerperal OR puerperium OR parturition OR expectant mother OR labo?r OR post?natal OR post?partal OR gestation* OR obstetric | exp delivery/ OR exp parturition/ OR exp pregnancy/ OR exp labor/  *(Include all subheadings)* | exp delivery, obstetric/ or exp parturition/ or exp pregnancy/ | (MH "Delivery, Obstetric+") or (MH "Labor+") or (MH "Pregnancy+") |

AND

*Frequency terms:*

| *Text* | *Embase* | *MEDLINE* | *CINAHL* |
| --- | --- | --- | --- |
| prevalence OR proportion OR percentage OR incidence OR rate* OR epidemiology | exp prevalence/ OR exp incidence/ OR epidemiology/  *(Include all subheadings)* | exp prevalence/ OR exp incidence/ OR epidemiology/ | (MH "Prevalence") OR (MH "Incidence") OR (MH "Epidemiology") |

AND

*Type of publications:*

| *Text* | *Embase* | *MEDLINE* | *CINAHL* |
| --- | --- | --- | --- |
| (((comprehensive* or integrative or systematic*) adj3 (bibliographic* or review* or literature)) or (meta-analy* or metaanaly* or "research synthesis" or ((information or data) adj3 synthesis) or (data adj2 extract*))).ti,ab. or (cinahl or (cochrane adj3 trial*) or embase or medline or psyclit or (psycinfo not "psycinfo database") or pubmed or scopus or "sociological abstracts" or "web of science").ab. or ("cochrane database of systematic reviews" or evidence report technology assessment or evidence report technology assessment summary).jn. or Evidence Report: Technology Assessment*.jn. or ((review adj5 (rationale or evidence)).ti,ab. and review.pt.) or meta-analysis as topic/ or Meta-Analysis.pt. | meta analysis/ OR exp systematic review/  *(Include all subheadings)* | meta analysis/ OR exp "Review Literature as Topic"/ | (MH "Meta analysis") OR (MH "Systematic review") |

AND each one of those below

| *Conditions* | *Free text* | *Embase* | *MEDLINE* | *CINAHL* | Free text CINAHL |
| --- | --- | --- | --- | --- | --- |
| *Pregnancy complications MeSH* |  | exp pregnancy complications/ OR exp pregnancy disorder/ OR exp labor complication/ | exp Pregnancy Complications/ or exp Puerperal Disorders/ or exp Obstetric Labor Complications/ | (MH "Labor Complications") or (MH "Pregnancy Complications") or (MH "Puerperal Disorders") |  |
| Hypertensive disorders | Hypertens* OR (gestational ADJ3 hypertens*) OR pre-eclampsia OR eclampsia OR HELLP OR ((h?emolysis ADJ2 "elevated liver enzymes" ADJ2 "low platelet count") AND syndrome) or (pregnancy?induced ADJ3 hyperten*) | Exp PREECLAMPSIA/ or exp "ECLAMPSIA AND PREECLAMPSIA"/or Exp ECLAMPSIA/ or exp maternal hypertension/ or HELLP syndrome/ or hypertension/ | Exp Hypertension, Pregnancy-Induced/ or Hypertension/ | (MH "Pregnancy-Induced Hypertension+") | Hypertens* OR (gestational N3 hypertens*) OR pre-eclampsia OR eclampsia OR HELLP OR  ((h#emolysis N2 "elevated liver enzymes" N2 "low platelet count") AND syndrome) or (pregnancy#induced N3 hyperten*) |
| *Ectopic pregnancy* | (Ectopic or abdom* or heterotopic or extra?uterine or tub* or ovar*) ADJ3 pregnanc* | Exp ectopic pregnancy/ | Exp Pregnancy, Ectopic/ | (MH "Pregnancy, Ectopic") | (Ectopic or abdom* or heterotopic or extra#uterine or tub* or ovar*) N3 pregnanc* |
| *Obstructed labour* | abnormal labo?r OR inertia uteri OR (labo?r AND obstruct*) OR uter* inertia OR (Dystoc* AND labo?r) OR dystoc* | exp dystocia/ | exp dystocia/ | (MH "Dystocia") | abnormal labo#r OR inertia uteri OR (labo#r AND obstruct*) OR uter* inertia OR (Dystoc* AND labo#r) OR dystoc* |
| *Placenta disorders –* *retained products of conception + accreta/increta/percreta placenta (morbidly adherent placenta) + placenta previa + placental abruption* | (Placenta* AND disorder*) OR (Retained AND (f?etal ADJ3 (tissue OR membrane*)) OR (Products ADJ3 Conception)) OR RPC OR retained placenta OR placental retention OR (Placenta* AND (ac?ret* OR incret* or percret*)) OR morbidly adherent placenta OR (Abnormal* AND placenta* implantation) OR placenta pr?evia OR ((ablatio* OR abruptio* OR detachment OR separation OR solutio*) AND placenta*) OR (retroplacental AND (h?ematoma OR h?emorrhage)) OR uteroplacental apoplexia | placenta disorder/ OR retained placenta/ OR placenta accreta/ OR placenta previa/ OR solution placentae/ | Placenta diseases/ OR Abruptio Placentae/ or Placenta Accreta/ or Placenta Previa/ OR Placenta, Retained/ | (MH "Placenta Diseases") OR (MH "Abruptio Placentae") OR (MH "Placenta Accreta") OR (MH "Placenta Praevia") OR (MH "Placenta, Retained") | (Placenta* AND disorder*) OR (Retained AND (f#etal N3 (tissue OR membrane*)) OR (Products N3 Conception)) OR RPC OR retained placenta OR placental retention OR (Placenta* AND (ac#ret* OR incret* or percret*)) OR morbidly adherent placenta OR  (Abnormal* AND placenta* implantation) OR placenta pr#evia OR ((ablatio* OR abruptio* OR detachment OR separation OR solutio*) AND placenta*) OR (retroplacental AND (h#ematoma OR h#emorrhage)) OR uteroplacental apoplexia |
| *Gestational trophoblastic disease* | ((Gestational Trophoblastic) ADJ3 (Disease OR neoplas* OR tumo?r)) OR (trophoblast* ADJ3 (neoplas* OR tumo?r)) OR trophoblastoma OR chorioadenoma OR choriocarcinoma OR hydatidiform mole OR invasive mole | Exp trophoblastic tumor/ | Exp Trophoblastic Neoplasms/ | (MH "Trophoblastic Neoplasms+") | ((Gestational Trophoblastic) N3 (Disease OR neoplas* OR tumo#r)) OR (trophoblast* N3 (neoplas* OR tumo#r)) OR trophoblastoma OR chorioadenoma OR choriocarcinoma OR hydatidiform mole OR invasive mole |
| *Postpartum haemorrhage* | (Post?part* adj4 (Fluxus or h?emorrhage or bleeding)) OR ((Fluxus or h?emorrhage or bleeding) adj4 Post?part*) or lochia or (uter* adj3 (bleeding or h?emorrhage OR trauma OR damag* OR lacerat* OR tear OR dehiscence)) or ((bleeding or h?emorrhage OR trauma OR damag* OR lacerat* OR tear OR dehiscence) adj3 uter*) | postpartum hemorrhage/ | Postpartum Hemorrhage/ | (MH "Postpartum Hemorrhage") | (Post#part* N4 (Fluxus or haemorrhage or hemorrhage or bleeding)) OR ((Fluxus or haemorrhage or hemorrhage or bleeding) N4 Post#part*) or lochia or (uter* N3 (bleeding or haemorrhage or hemorrhage OR trauma OR damag* OR lacerat* OR tear OR dehiscence)) or ((bleeding or haemorrhage OR hemorrhage OR trauma OR damag* OR lacerat* OR tear OR dehiscence) N3 uter*) |
| *Thrombotic* | Phlebothrombosis OR (venous ADJ3 thrombosis) OR (thrombosis ADJ3 venous) OR vena thrombosis or vein thrombosis or Thrombophlebitis or phlebitis or venous thromboembolism or VTE | Exp vein thrombosis/ or thrombophlebitis/ or Thrombosis/ | exp Venous Thrombosis/ or Thrombosis/ | (MH "Venous Thrombosis+") or (MH "Thrombosis") | Phlebothrombosis OR (venous N3 thrombosis) OR (thrombosis N3 venous) OR vena thrombosis or vein thrombosis or Thrombophlebitis or phlebitis |
| *Nausea and vomiting of pregnancy* | ((Nausea ADJ2 vomit*) AND pregnancy) OR NVP OR gestation* vomit* OR hyperemesis gravidarum or morning sickness | "nausea and vomiting"/ or nausea/ or vomiting/ or morning sickness/ or hyperemesis gravidarum/ | nausea/ or vomiting/ or exp Morning Sickness/ | (MH "Hyperemesis Gravidarum") | ((Nausea N2 vomit*) AND pregnancy) OR NVP OR gestation* vomit* OR hyperemesis gravidarum or morning sickness |
| *Cholestasis of pregnancy* | ((cholestasis or bile stasis) ADJ3 pregnancy) OR obstetric cholestasis | intrahepatic cholestasis/ | Cholestasis, Intrahepatic/ | (MH "Cholestasis, Intrahepatic ") | ((cholestasis or bile stasis) N3 pregnancy) OR obstetric cholestasis |
| *Peripartum cardiomyopathy* | ((myocardiopathy or cardiomyopathy) ADJ2 (peri?partum or puerperal or post?partum)) OR ((peri?partum or puerperal or post?partum) ADJ2 (myocardiopathy or cardiomyopathy)) OR PPCM | peripartum cardiomyopathy/ | Exp cardiomyopathies/ | (MH "Myocardial Diseases+") | ((myocardiopathy or cardiomyopathy) N2 (peri#partum or puerperal or post#partum)) OR ((peri#partum or puerperal or post#partum) N2 (myocardiopathy or cardiomyopathy)) OR PPCM |
| *Mirror syndrome* | ((Mirror Or Ballantyne) AND Syndrome) or triple ?edema or ((f?et* hydrops or hydrops f?etalis or neonatal hydrops) and pre?eclampsia) | (fetus hydrops/ and preeclampsia/) or (fetus hydrops/ and edema/) | (Hydrops Fetalis/ and edema/) or (Hydrops Fetalis/ and Pre-Eclampsia/) | (MH "Mirror Syndrome") | ((Mirror Or Ballantyne) AND Syndrome) or triple #edema or ((f#et* hydrops or hydrops f#etalis or neonatal hydrops) and pre#eclampsia) |
| *Gestational diabetes* | Diabetes mellitus gravidarum or ((Gestation* or pregnancy) ADJ3 diabet*) OR (diabet* ADJ3 (Gestation* or pregnancy)) | Exp pregnancy diabetes mellitus/ or endocrine diseases/ or  diabetes mellitus/ or pancreas islet disease/ | Exp "pregnancy in Diabetics"/ or exp Diabetes, Gestational/ or Endocrine System Diseases/ or Diabetes Mellitus/ | (MH "Pregnancy in Diabetes+") or (MH "Diabetes Mellitus") or MH("Diabetes Mellitus, Type 1") or MH("Diabetes Mellitus, Type 2") or (MH "Endocrine Diseases") | Diabetes mellitus gravidarum or ((Gestation* or pregnancy) N3 diabet*) OR (diabet* N3 (Gestation* or pregnancy)) |
| *Postpartum thyroiditis* | post?partum thyr?oiditis | postpartum thyroiditis/ | postpartum thyroiditis/ | (MH "Thyroiditis+") | post#partum thyr#oiditis |
| *Intentional self-harm* | Suicide or (Suicid* and (ideation or attempt or behavio?r)) or self?poisoning or Self?harm or self?injur* or self?mutilation or self?wounding or (self?inflicted ADJ3 (injur* or harm or wound*)) or auto?mutilation | Exp suicidal behavior/ or automutilation/ | Exp Self-Injurious Behavior/ | (MH "Self-Injurious Behavior") or (MH "Injuries, Self-Inflicted") | Suicide or (Suicid* and (ideation or attempt or behavio#r)) or self#poisoning or Self#harm or self#injur* or self#mutilation or self#wounding or (self#inflicted N3 (injur* or harm or wound*)) or auto#mutilation |
| *Mastitis/breast abscess* | (Breast and (infection or inflammation or abscess)) or mastitis or comedomastitis or (mammary and (infection or abscess)) | Exp mastitis/ | Exp Mastitis/ | (MH "Lactation Disorders+") | (Breast and (infection or inflammation or abscess)) or mastitis or comedomastitis or (mammary and (infection or abscess)) |
| *Chorioamnionitis* | Chorioamnionitis or amnionitis or ((intra?amniotic or amnion or chorion or f?etal membrane*) ADJ3 infection) or (infection ADJ3 (intra?amniotic or amnion or chorion or f?etal membrane*)) | Exp Chorioamnionitis/ | Exp Chorioamnionitis/ | (MH "Chorioamnionitis") | Chorioamnionitis or amnionitis or ((intra#amniotic or amnion or chorion or f#etal membrane*) N3 infection) or (infection N3 (intra#amniotic or amnion or chorion or f#etal membrane*)) |
| *Aspiration pneumonitis* | ((Aspiration* pneumoni*) and anesthes*) or (Mendelson* ADJ3 syndrome) or (syndrome ADJ3 Mendelson) | Exp aspiration pneumonia/ | Exp Pneumonia, Aspiration/ | (MH "Pneumonia, Aspiration") | ((Aspiration* pneumoni*) and anesthes*) or (Mendelson* N3 syndrome) or (syndrome N3 Mendelson) |
| *Cerebral anoxia* | (Brain or cerebral) and (anoxia or hypoxia or asphyxia) | Brain hypoxia/ | Exp Hypoxia, Brain/ | (MH "Hypoxia, Brain+") | (Brain or cerebral) and (anoxia or hypoxia or asphyxia) |
| *Perineal tear (3rd or 4th degree) + vaginal wall/perineal laceration + urethral tear/damage* | (((Vagin* or perine* or urethra* or vulva*) ADJ3 (laceration* or tear* or damag* or injur* or trauma* or lesion*)) or ((laceration* or tear* or damag* or injur* or trauma* or lesion*) ADJ3 (Vagin* or perine* or urethra* or vulva*))) AND (labo?r or delivery) | (vaginal injury/ or perineum injury/ or urethra injury/ or (vulva/ and injury/)) and (vaginal delivery/ or labor complication/) | ("Wounds and Injuries"/ and (Perineum/ or vagina/ or Urethra/ or vulva/)) | (MH "Wounds and Injuries") and ((MH "Perineum" or (MH "Vagina") or (MH "Urethra") or (MH "Vulva")) | (((Vagin* or perine* or urethra* or vulva*) N3 (laceration* or tear* or damag* or injur* or trauma* or lesion*)) or ((laceration* or tear* or damag* or injur* or trauma* or lesion*) N3 (Vagin* or perine* or urethra* or vulva*))) AND (labo#r or delivery) |
| *Vulval hematoma* | ((Vulva* ADJ3 H?ematoma) Or (H?ematoma ADJ3 Vulva*)) and (delivery or labo?r) | (Hematoma/ or traumatic hematoma/) and (vaginal delivery/ or labor complication/) | Hematoma/ and vulva/ | (MH "Hematoma") and (MH "vulva") | ((Vulva* N3 H#ematoma) Or (H#ematoma N3 Vulva*)) and (delivery or labo#r) |
| *Episiotomy infection* | Episiotomy and (infect* or abscess) | Episiotomy/ and (infection/ or labor complication/) | Episiotomy/ and (exp Wound Infection/ or Obstetric Labor Complications/) | (MH "Episiotomy") AND ((MH "Wound Infection+") or (MH "Labor Complications")) | Episiotomy and (infect* or abscess) |
| *Tetanus* | Tetanus or Clostridium tetani infection | Tetanus/ | Tetanus/ | (MH "Tetanus") | Tetanus or Clostridium tetani infection |
| *Sepsis* | ((puerper* or post?partum or maternal or pregnan*) ADJ5 (sepsis or infect* or septic or fever or pyrexi* or activated protein C or toxic shock or septic?em* or endometritis or metritis or endomyometritis or endoparametritis or membranitis)) or ((sepsis or infect* or septic shock or fever or pyrexi* or activated protein C or toxic shock) ADJ5 (puerper* or post?partum or maternal or pregnan*)) or placentitis or streptococcus | puerperal infection/ or exp systemic inflammatory response syndrome/ | puerperal infection/ or Pregnancy Complications, Infectious/ or exp Systemic Inflammatory Response Syndrome/ | (MH "Pregnancy Complications, Infectious+") or (MH "Systemic Inflammatory Response Syndrome+") | ((puerper* or post?partum or maternal or pregnan*) ADJ5 (sepsis or infect* or septic or fever or pyrexi* or activated protein C or toxic shock or septic?em* or endometritis or metritis or endomyometritis or endoparametritis or membranitis)) or ((sepsis or infect* or septic shock or fever or pyrexi* or activated protein C or toxic shock) ADJ5 (puerper* or post?partum or maternal or pregnan*)) or placentitis or streptococcus |
| *Urinary tract infection (UTI)* | Urin* tract infection or genitourinary tract infection* or urin* infection* or urologic infection or UTI or tractus urogenitalis infection or kidney infection or pyelonephritis | exp urinary tract infection/ or exp pyelonephritis/ | Exp Urinary Tract Infections/ or exp Pyelonephritis/ | (MH "Urinary Tract Infections+") or (MH "Pyelonephritis") | Urin* tract infection or genitourinary tract infection* or urin* infection* or urologic infection or UTI or tractus urogenitalis infection or kidney infection or pyelonephritis |
| *Cesarean delivery* |  |  |  |  |  |
| *Uterine perforation* | (Uter* AND (perforat* OR rupture*)) AND (c?esar#an OR abdominal operation OR abdominal delivery OR post? c?esar#an OR fefectomy OR sectio caesarea OR cesarotomy or c-section*) | (uterus perforation/ OR uterus rupture/) AND cesarean section/ | Exp Uterine Rupture/ and exp Cesarean Section/ | (MH "Uterine Rupture") and (MH "Cesarean Section+") | (Uter* AND (perforat* OR rupture*)) AND (c#esar#an OR abdominal operation OR abdominal delivery OR post# c#esar#an OR fefectomy OR sectio caesarea OR cesarotomy or c-section*) |
| *Postpartum inversion of uterus* | Uter* and inver* | uterus inversion/ | Uterine Inversion/ | (MH "Uterine Inversion") | Uter* and inver* |
| *Cesarean delivery wound infection* | (((Surgical or surgery) AND (site or incision)) or wound or incision) AND infect* AND (c?esar#an OR abdominal operation OR abdominal delivery OR post? c?esar#an OR fefectomy OR sectio caesarea OR cesarotomy or c-section*) | (wound infection/ OR surgical infection/ OR postoperative infection/) AND cesarean section/ | Exp wound infection/ and exp Cesarean Section/ | (MH "wound infection+") and (MH "Cesarean Section+") | (((Surgical or surgery) AND (site or incision)) or wound or incision) AND infect* AND (c#esar#an OR abdominal operation OR abdominal delivery OR post# c#esar#an OR fefectomy OR sectio caesarea OR cesarotomy or c-section*) |
| *Postoperative ileus/bowel obstruction* | (Ileus or ((intestin* or bowel or colon) AND (obstruct* or occlusion))) AND (c?esar#an OR abdominal operation OR abdominal delivery OR post? c?esar#an OR fefectomy OR sectio caesarea OR cesarotomy or c-section*) | exp intestine obstruction/ and cesarean section/ | (intestine obstruction/ or ileus/) and (exp Cesarean Section/) | (MH "Intestinal Obstruction") and (MH "Cesarean Section+") | (Ileus or ((intestin* or bowel or colon) AND (obstruct* or occlusion))) AND (c#esar#an OR abdominal operation OR abdominal delivery OR post# c#esar#an OR fefectomy OR sectio caesarea OR cesarotomy or c-section*) |
| *Ovarian hyperstimulation syndrome* | (Ovar* and hyperstimulation and syndrome) or OHSS | ovary hyperstimulation/ | Ovarian Hyperstimulation Syndrome/ | (MH "Ovarian Hyperstimulation Syndrome") | (Ovar* and hyperstimulation and syndrome) or OHSS |
| *Nosocomial or hospital acquired infection (UTI, C diff, pneumonia)* | ((Hospital?acquired OR hospital?associated OR hospital OR ward OR nosocomial OR health?care OR heath care?associated) ADJ4 infection*) OR (infection* ADJ4 (Hospital?acquired OR hospital?associated OR hospital OR ward OR nosocomial OR health?care OR heath care?associated)) | Hospital infection/ | Exp Cross Infection/ or ((exp Health Facilities/ or exp Health Services/) and exp infection/) | (MH "Cross Infection") or (((MH "Health Facilities+") or (MH "Health Services+")) and (MH "Infection+")) | ((Hospital#acquired OR hospital#associated OR hospital OR ward OR nosocomial OR health#care OR heath care#associated) N4 infection*) OR (infection* N4 (Hospital#acquired OR hospital#associated OR hospital OR ward OR nosocomial OR health#care OR heath care#associated)) |
| *Indirect conditions* | | |  |  |  |
| *HIV/AIDS* | HIV OR AIDS OR Human immune?deficiency virus OR acquired immune deficiency syndrome | Exp Human immunodeficiency virus/ | Exp HIV/ | (MH "Human Immunodeficiency Virus+") | HIV OR AIDS OR Human immune#deficiency virus OR acquired immune deficiency syndrome |
| *Tuberculosis mycobacterium* | Tuberculosis or human tubercle bacill* or Koch* bacillus or mycobacterium tuberculosum | Mycobacterium tuberculosis/ or exp tuberculosis/ | Exp tuberculosis/ or Mycobacterium Infections/ or Mycobacterium tuberculosis/ | (MH "Mycobacterium Infections+") or (MH "Mycobacterium tuberculosis") | Tuberculosis or human tubercle bacill* or Koch* bacillus or mycobacterium tuberculosum |
| *Malaria* | Malaria or swamp fever or paludism or plasmodium or P* falciparum or P* vivax or P* ovale or P* malariae | Exp malaria/ | Exp Malaria/ | (MH "Malaria") | Malaria or swamp fever or paludism or plasmodium or P* falciparum or P* vivax or P* ovale or P* malariae |
| *Sexually transmitted infection (STI)* | STI or ((sexually transmitted or vener?al) AND (infection* or disease* or condition*)) or condyl* a?cuminat* or ((penis or vulva*) and condyl*) or verruca a?cuminat* or Chlamydia or HPV or ((human or infection) and papillomavirus) or wart* or herpes or syphili* or T* pallidum or lues or chancre or tabes dorsalis | Exp sexually transmitted disease/ or Chlamydia trachomatis/ or papillomavirus infection/ | Exp Sexually Transmitted Diseases/ or exp Papillomavirus Infections/ | (MH "Sexually Transmitted Diseases+") or (MH "Papillomavirus Infections+") | STI or ((sexually transmitted or vener#al) AND (infection* or disease* or condition*)) or condyl* a#cuminat* or ((penis or vulva*) and condyl*) or verruca a#cuminat* or Chlamydia or HPV or ((human or infection) and papillomavirus) or wart* or herpes or syphili* or T* pallidum or lues or chancre or tabes dorsalis |
| *Candidiasis* | Candidiasis or candidamycosis or candidosis | Candidiasis/ or vagina candidiasis/ or genital candidiasis/ | Candidiasis/ or Candidiasis, Vulvovaginal/ | (MH "Candidiasis") or (MH "Candidiasis, Vulvovaginal") | Candidiasis or candidamycosis or candidosis |
| *Influenza* | Influenza or flu or epidemic* bronchitis | Exp Influenza/ | Influenza, Human/ | (MH "Influenza, Human+") | Influenza or flu or epidemic* bronchitis |
| *Pneumonia* | Pneumoni* or ((lung or pulmon*) and (infect* or inflammat*)) or lobitis or peripneumonia or pleuropneumoni* or (pneumonic and (lung or pleurisy or pleuritis)) | Exp pneumonia/ | Exp pneumonia/ | (MH "Pneumonia+") | Pneumoni* or ((lung or pulmon*) and (infect* or inflammat*)) or lobitis or peripneumonia or pleuropneumoni* or (pneumonic and (lung or pleurisy or pleuritis)) |
| *Hepatitis* | Hepatitis or (vir* liver ADJ3 (disease or infect*)) or HAV or HBV or HCV or HEV | Exp hepatitis/ | Exp hepatitis/ | (MH "Hepatitis+") | Hepatitis or (vir* liver N3 (disease or infect*)) or HAV or HBV or HCV or HEV |
| *Varicella* | Chicken?pox or varicella* or varicellovirus or zoster or vzv | Chickenpox/ or exp herpes zoster/ or exp Varicella zoster virus/ | Chickenpox/ or exp Herpes Zoster/ | (MH "Chickenpox") or (MH "Herpes Zoster+") | Chicken#pox or varicella* or varicellovirus or zoster or vzv |
| *Cholera* | Cholera or Vibrio cholerae | Cholera/ | Cholera/ | (MH "Cholera") | Cholera or Vibrio cholerae |
| *Skin (all)* | Dermatos#s or Eczema or Atopic dermatitis or Prurigo or pruritus or (((Pruritic Urticarial Papules) ADJ3 Plaques) ADJ3 Pregnancy) or PUPP or ((Polymorphic Eruption) ADJ3 Pregnancy) or PEP or Linea nigra or pregnancy line or Acne or Psoriasis | skin disease/ or Dermatitis/ or Exp [atopic dermatitis](http://ovidsp.tx.ovid.com/sp-3.18.0b/ovidweb.cgi?S=KLLLFPMMGJDDFEGNNCJKPGOBFAJDAA00&Controlled+Vocabulary=thes+atopic+dermatitis&)/ or Exp eczema/ or Exp acne/ or Exp Psoriasis/ or Exp pruritus/ or [hyperpigmentation](http://ovidsp.tx.ovid.com/sp-3.18.0b/ovidweb.cgi?&Controlled+Vocabulary=Mapping%7c3&Return=mapping&S=KLLLFPMMGJDDFEGNNCJKPGOBFAJDAA00)/ or skin pigmentation/ or skin disorder/ | skin diseases/ or exp Dermatitis/ or exp Acneiform Eruptions/ or exp Pruritus/ or Hyperpigmentation/ or Pigmentation Disorders/ or exp Psoriasis/ | (MH "skin diseases") or (MH "Dermatitis+") or (MH "Acneiform Eruptions+") or (MH "Pruritus+") or (MH "Hyperpigmentation") or (MH "Pigmentation Disorders") or (MH "Psoriasis+") | Dermatos#s or Eczema or Atopic dermatitis or Prurigo or pruritus or (((Pruritic Urticarial Papules) N3 Plaques) N3 Pregnancy) or PUPP or ((Polymorphic Eruption) N3 Pregnancy) or PEP or Linea nigra or pregnancy line or Acne or Psoriasis |
| *Anemia* | An?emia or ((sickle?cell) AND (disease or an?emia)) or [drepanocytemia](http://ovidsp.tx.ovid.com/sp-3.18.0b/ovidweb.cgi?S=ONFGFPPKJIDDEEAPNCJKHHGCFGEAAA00&Controlled+Vocabulary=thes+drepanocytemia&) or drepanocytic an?emia or drepanocytosis or [h?emoglobin SS](http://ovidsp.tx.ovid.com/sp-3.18.0b/ovidweb.cgi?S=ONFGFPPKJIDDEEAPNCJKHHGCFGEAAA00&Controlled+Vocabulary=thes+haemoglobin+SS&) or Hb SS or [meniscocytosis](http://ovidsp.tx.ovid.com/sp-3.18.0b/ovidweb.cgi?S=ONFGFPPKJIDDEEAPNCJKHHGCFGEAAA00&Controlled+Vocabulary=thes+meniscocytosis&) or SS disease or sickle an?emia or tha?la?s?emia | Exp anemia/ or exp thalassaemia/ or exp sickle cell anemia/ | Exp anemia/ | (MH "Anemia") | An#emia or ((sickle#cell) AND (disease or an#emia)) or [drepanocytemia](http://ovidsp.tx.ovid.com/sp-3.18.0b/ovidweb.cgi?S=ONFGFPPKJIDDEEAPNCJKHHGCFGEAAA00&Controlled+Vocabulary=thes+drepanocytemia&) or drepanocytic an#emia or drepanocytosis or [h#emoglobin SS](http://ovidsp.tx.ovid.com/sp-3.18.0b/ovidweb.cgi?S=ONFGFPPKJIDDEEAPNCJKHHGCFGEAAA00&Controlled+Vocabulary=thes+haemoglobin+SS&) or Hb SS or [meniscocytosis](http://ovidsp.tx.ovid.com/sp-3.18.0b/ovidweb.cgi?S=ONFGFPPKJIDDEEAPNCJKHHGCFGEAAA00&Controlled+Vocabulary=thes+meniscocytosis&) or SS disease or sickle an#emia or tha#la#s#emia |
| *Idiopathic thrombocytopenic purpura (ITP)* | autoimmune thrombocytop?eni* or immune thrombocytop?eni* or idiopathic thrombocytop?eni* or thrombocytop?eni* purpura or ITP or Werlhof disease or morbus werlhof | idiopathic thrombocytopenic purpura/ | Purpura, Thrombocytopenic, Idiopathic/ or Purpura, Thrombocytopenic/ | (MH "Purpura, Thrombocytopenic") | autoimmune thrombocytop#eni* or immune thrombocytop#eni* or idiopathic thrombocytop#eni* or thrombocytop#eni* purpura or ITP or Werlhof disease or morbus werlhof |
| *Hypothyroidism* | Hypothyr?oid* or hypothyr?osis or (thyr?oid* AND (deficiency or failure or insufficiency)) | exp hypothyroidism/ or Exp myxedema/ or Exp subclinical hypothyroidism/ | Exp Hypothyroidism/ | (MH "Hypothyroidism+") | Hypothyr#oid* or hypothyr#osis or (thyr#oid* AND (deficiency or failure or insufficiency)) |
| *Hyperthyroidism* | Hyperthyr?oid* or hypothyr?osis or (thyr?oid* AND hyperfunction) or hyperthyr?oid function | exp hyperthyroidism/ | Exp Hyperthyroidism/ | (MH "Hyperthyroidism+") | Hyperthyr#oid* or hypothyr#osis or (thyr#oid* AND hyperfunction) or hyperthyr#oid function |
| *Hyperparathyroidism* | Hyperparathyroid* or hyper parathyroidism or (parathyroid and (hyperfuction or hypertroph*)) | hyperparathyroidism/ | Exp hyperparathyroidism/ | (MH "hyperparathyroidism+") | Hyperparathyroid* or hyper parathyroidism or (parathyroid and (hyperfuction or hypertroph*)) |
| *Acquired and congenital structural heart disease (including valvular heart disease)* | ((Cardiac or heart) ADJ3 (anomaly or deficiency or deformity or malformation)) or ((anomaly or deficiency or deformity or malformation) ADJ3 (Cardiac or heart)) or structur* heart disease or structur* cardiac disease or cardiopathy or congenital heart disease or ((valv* heart or cardiac valv* or heart valv* or valv* cardiac) ADJ3 (disease* or lesion* or defect* or abnormal*)) or ((disease* or lesion* or defect* or abnormal*) ADJ3 (valv* heart or cardiac valv* or heart valv* or valv* cardiac)) or atri* sept* defect or ASD or patent foramen ovale or PFO or (coarctation and aorta) or ((stenosis or insufficiency or prolapse) and (aort* or mitral or pulmonar or tricuspid)) or valvulopath* | Heart disease/ or Exp congenital heart disease/ or Exp valvular heart disease/ | Heart diseases/ or exp Heart Defects, Congenital/ or exp Heart Valve Diseases/ | (MH "Heart diseases") OR (MH "Heart Defects, Congenital+") OR (MH "Heart Valve Diseases+") | ((Cardiac or heart) N3 (anomaly or deficiency or deformity or malformation)) or ((anomaly or deficiency or deformity or malformation) N3 (Cardiac or heart)) or structur* heart disease or structur* cardiac disease or cardiopathy or congenital heart disease or ((valv* heart or cardiac valv* or heart valv* or valv* cardiac) N3 (disease* or lesion* or defect* or abnormal*)) or ((disease* or lesion* or defect* or abnormal*) N3 (valv* heart or cardiac valv* or heart valv* or valv* cardiac)) or atri* sept* defect or ASD or patent foramen ovale or PFO or (coarctation and aorta) or ((stenosis or insufficiency or prolapse) and (aort* or mitral or pulmonar or tricuspid)) or valvulopath* |
| *Aortic dissection* | Aort* ADJ3 dissection or dissection ADJ3 aort* | Aorta dissection/ | Aortic diseases/ | (MH "Aortic Dissections") | Aort* N3 dissection or dissection N3 aort* |
| *Arrhythmia* | Arrhythmia or dysrhythmia or dysrhythmia or ectopic heart rhythm or (ectopic ADJ2 rhythm) or ectopic beat or aberrant conduction or ectopic ventricle contraction or rhythm disorder or bradycardia or bradyarrhythmia or bradycardy or bradycardia or hypoarrhythmia or brachycardia or (heart rate ADJ2 (low or high)) or ((low or high) ADJ2 heart rate) or tachycardia or tachyarrhythmia or heart hyperfunction or palpitation or atri* fibrillation or ventric* fibrillation | Exp heart arrhythmia/ | Exp Arrhythmias, Cardiac/ | (MH "Arrhythmia+") | Arrhythmia or dysrhythmia or dysrhythmia or ectopic heart rhythm or (ectopic N2 rhythm) or ectopic beat or aberrant conduction or ectopic ventricle contraction or rhythm disorder or bradycardia or bradyarrhythmia or bradycardy or bradycardia or hypoarrhythmia or brachycardia or (heart rate N2 (low or high)) or ((low or high) N2 heart rate) or tachycardia or tachyarrhythmia or heart hyperfunction or palpitation or atri* fibrillation or ventric* fibrillation |
| *Cardiomyopathy* | Cardiomyopath* or heart myopathy* or myocardiopath* or primary myocard* disease* | Exp cardiomyopathy/ | Exp Cardiomyopathies/ | (MH "Myocardial Diseases+") | Cardiomyopath* or heart myopathy* or myocardiopath* or primary myocard* disease* |
| *Asthma* | Asthma* or allerg* lung or lung allerg* | Exp asthma/ or respiratory diseases/ | Exp asthma/ or Respiratory Tract Diseases/ | (MH "asthma+") or (MH " Respiratory Tract Diseases") | Asthma* or allerg* lung or lung allerg* |
| *Obstructive sleep apnea* | sleep disordered breath* or nocturnal apn?ea or sleep apn?ea or osa or osas or obesity hypoventilation syndrome or upper airway resistance syndrome | Exp sleep disordered breathing/ | Exp Sleep Apnea, Obstructive/ | (MH "Sleep Apnea, Obstructive") | sleep disordered breath* or nocturnal apn#ea or sleep apn#ea or osa or osas or obesity hypoventilation syndrome or upper airway resistance syndrome |
| *Pulmonary embolism* | Lung embol* or pulmonar* embol* or lung microembol* or pulmonar* microembol* or pulmonar* thromboembol* or lung thromboembol* or PTE or PE OR amniotic fluid embol* | lung embolism/ OR amnion fluid embolism/ | Exp Pulmonary Embolism/ or Embolism, Amniotic Fluid/ | (MH "Pulmonary Embolism") or (MH "Embolism, Amniotic Fluid") | Lung embol* or pulmonar* embol* or lung microembol* or pulmonar* microembol* or pulmonar* thromboembol* or lung thromboembol* or PTE or PE |
| *Anal fissure* | (Anal or anus or anorectal or ani or ano) AND fissur* | anus fissure/ | Fissure in Ano/ | (MH "Fissure in Ano") | (Anal or anus or anorectal or ani or ano) AND fissur* |
| *Hemorrhoids* | H?emorrhoid* | hemorrhoid/ | Hemorrhoids/ | (MH "Hemorrhoids") | H#emorrhoid* |
| *Cholecystitis* | Cholangiocholecystitis or gall?bladder infection or gall?bladder inflammation or cholecystitis | Exp cholecystitis/ | Exp Cholecystitis/ | (MH "Cholecystitis+") | Cholangiocholecystitis or gall#bladder infection or gall#bladder inflammation or cholecystitis |
| *Cholylethiasis* | (Bil* and (lithiasis or lithogenicity or calcul*)) or (gall?bladder and (stone* or calcul*)) or gallstone disease or cholylethiasis or cholecystolithiasis or cholethiasis or cholelithiasis | Exp Cholelithiasis/ | Exp Cholecystolithiasis/ | (MH "Cholelithiasis") | (Bil* and (lithiasis or lithogenicity or calcul*)) or (gall#bladder and (stone* or calcul*)) or gallstone disease or cholylethiasis or cholecystolithiasis or cholethiasis or cholelithiasis |
| *Gastroesophageal reflux disease (GERD)* | ((gastro?esophageal or gastro?oesophageal or ?esophag* or ?esophagogastric or cardio?esophageal or gastro?esophagus) and (reflux or regurgitation or reflex)) or GERD or GORD | Exp gastroesophageal reflux/ | Exp Gastroesophageal Reflux/ | (MH "Gastroesophageal Reflux") | ((gastro#esophageal or gastro#oesophageal or #esophag* or #esophagogastric or cardio#esophageal or gastro#esophagus) and (reflux or regurgitation or reflex)) or GERD or GORD |
| *Inflammatory bowel disease (IBD)* | Inflammatory Bowel Disease* or IBD or ulcerative colitis or colitis ulcerative or colitis ulcerosa or ulcerative procto?colitis or ulcerative colorectitis or ulcerous colitis or mucosal colitis or colon ulceration or UC or cleron disease or Crohn* disease or (regional* and (enteritis or enterocolitis)) or morbus crohn or CD | Exp inflammatory bowel disease/ | Exp Inflammatory Bowel Diseases/ | (MH "Inflammatory Bowel Diseases+") | Inflammatory Bowel Disease* or IBD or ulcerative colitis or colitis ulcerative or colitis ulcerosa or ulcerative procto#colitis or ulcerative colorectitis or ulcerous colitis or mucosal colitis or colon ulceration or UC or cleron disease or Crohn* disease or (regional* and (enteritis or enterocolitis)) or morbus crohn or CD |
| *Bell's palsy* | Bell* Palsy or (facial AND (palsy or neuropath* or paralysis)) or VII nerve palsy | Exp Bell palsy/ | Bell Palsy/ | (MH "Bell palsy") | Bell* Palsy or (facial AND (palsy or neuropath* or paralysis)) or VII nerve palsy |
| *Carpal tunnel syndrome* | carpal canal syndrome or carpal tunnel compression or carpal tunnel syndrome or CTS or median neuropathy | carpal tunnel syndrome/ | carpal tunnel syndrome/ | (MH "carpal tunnel syndrome") | carpal canal syndrome or carpal tunnel compression or carpal tunnel syndrome or CTS or median neuropathy |
| *Migraine* | Migraine or (headache and migrainous) or hemicranias or status hemicranicus or recurrent headache* or unilateral headache* or hemicran* headache* | Exp migraine/ | Exp Migraine Disorders/ | (MH "Migraine") | Migraine or (headache and migrainous) or hemicranias or status hemicranicus or recurrent headache* or unilateral headache* or hemicran* headache* |
| *Multiple sclerosis* | ((Multiple or disseminated or insular or multiplex) AND sclerosis) or ms or chariot disease | Multiple sclerosis/ | Exp Multiple sclerosis/ | (MH "Multiple sclerosis+") | ((Multiple or disseminated or insular or multiplex) AND sclerosis) or ms or chariot disease |
| *Restless leg syndrome* | Restless leg* or anxietas tibiarum or RLS | restless legs syndrome/ | Restless Legs Syndrome/ | (MH "Restless Legs") | Restless leg* or anxietas tibiarum or RLS |
| *Seizure disorder (excluding eclampsia)* | Fit or convuls* or electroconvulsion or seizure* | Exp seizure/ or exp convulsion/ | Seizures/ | (MH "Seizures+") | Fit or convuls* or electroconvulsion or seizure* |
| *Arthritis (inflammatory, noninflammatory and rheumatoid)* | Arthritis or monoarthritis or oligoarthritis or arthrochondritis or arthrosynovitis or joint inflammation or osteoarthritis or poly?arthritis or beauvais disease or rheumarthritis or (rheumatism and articular) or RA | Exp arthritis/ | Exp Arthritis/ | (MH "Arthritis+") | Arthritis or monoarthritis or oligoarthritis or arthrochondritis or arthrosynovitis or joint inflammation or osteoarthritis or poly#arthritis or beauvais disease or rheumarthritis or (rheumatism and articular) or RA |
| *Ankylosing spondylitis* | (Ankylos* or ankylating or ankylopoietic*) and (spondylitis or spondylarthritis or spin* or spondylarthrosis or vertebral) or Bechterew disease or bekhterev disease or morbus bechterew | ankylosing spondylitis/ | Spondylitis, Ankylosing/ | (MH "Spondylitis, Ankylosing") | (Ankylos* or ankylating or ankylopoietic*) and (spondylitis or spondylarthritis or spin* or spondylarthrosis or vertebral) or Bechterew disease or bekhterev disease or morbus bechterew |
| *Systemic lupus erythematosus (SLE)* | Lupus or dermatovisceritism or lupovisceritis or erythematodes visceralis or osler libman sacks disease or sle | systemic lupus erythematosus/ | Exp Lupus Erythematosus, Systemic/ | (MH "Lupus Erythematosus, Systemic+") | Lupus or dermatovisceritism or lupovisceritis or erythematodes visceralis or osler libman sacks disease or sle |
| *Back pain* | ((Back or loin or low?back or lumba* or lumbosacral or lumbosacroiliac or flank) ADJ3 (pain or ache or strain)) or ((pain or ache or strain) ADJ3 (Back or loin or low?back or lumba* or lumbosacral or lumbosacroiliac or flank)) or dorsalgia or lumbalgia or lumbalgesia or backpain or backache or lumbago or (lumb* ADJ2 syndrome) or (syndrome ADJ2 lumb*) or lumbodynia | Exp backache/ or musculoskeletal diseases/ or musculoskeletal pain/ | Exp Back pain/ or musculoskeletal diseases/ or musculoskeletal pain/ | (MH "Back pain+") or (MH "musculoskeletal diseases") or (MH "muscle pain") | ((Back or loin or low#back or lumba* or lumbosacral or lumbosacroiliac or flank) N3 (pain or ache or strain)) or ((pain or ache or strain) N3 (Back or loin or low#back or lumba* or lumbosacral or lumbosacroiliac or flank)) or dorsalgia or lumbalgia or lumbalgesia or backpain or backache or lumbago or (lumb* N2 syndrome) or (syndrome N2 lumb*) or lumbodynia |
| *Acute and chronic kidney disease* | ((disease* or disorder* or patholog*) and (kidney or renal)) or nephropathy or perinephritis | Exp kidney disease/ | Exp Kidney Diseases/ | (MH "Kidney Diseases+") | ((disease* or disorder* or patholog*) and (kidney or renal)) or nephropathy or perinephritis |
| *Incontinence* | ((bladder or urin* or mixed or stress or urge*) and (incontinence or incontinentia or leakage or wetting or overactive)) | Exp urine incontinence/ | Exp Urinary Incontinence/ | (MH "Urinary Incontinence") | ((bladder or urin* or mixed or stress or urge*) and (incontinence or incontinentia or leakage or wetting or overactive)) |
| *Uterine/uterovaginal prolapse (including cystocele)* | (Prolaps* adj3 (uter* or utero?vaginal or bladder or genital or pelvic organ* or genitourinary or urogenital or vagina*)) or ((uter* or utero?vaginal or bladder or genital or pelvic organ* or genitourinary or urogenital or vagina*) adj3 prolaps*) or procidentia or (descensus and uter*) or bladder protusion or ((cystic or bladder) adj3 hernia) | Exp pelvic organ prolapse/ | Exp pelvic organ prolapse/ | (MH "Pelvic Organ Prolapse+") | (Prolaps* N3 (uter* or utero#vaginal or bladder or genital or pelvic organ* or genitourinary or urogenital or vagina*)) or ((uter* or utero#vaginal or bladder or genital or pelvic organ* or genitourinary or urogenital or vagina*) N3 prolaps*) or procidentia or (descensus and uter*) or bladder protusion or ((cystic or bladder) N3 hernia) |
| *Rectovaginal fistula/vesicovaginal fistula* | (Fistula adj3 (rectovaginalis or rectal vaginal or recto?vaginal or rectum vagina or bladder vaginal or vesicovaginalis or vagino?vesical or vesico?vagina* or cysto?vaginal)) or ((rectovaginalis or rectal vaginal or recto?vaginal or rectum vagina or bladder vaginal or vesicovaginalis or vagino?vesical or vesico?vagina*) adj3 fistula) or VVF or RVF | rectovaginal fistula/ or cystovaginal fistula/ | Exp Vaginal Fistula/ | (MH "Vaginal Fistula+") | (Fistula N3 (rectovaginalis or rectal vaginal or recto#vaginal or rectum vagina or bladder vaginal or vesicovaginalis or vagino#vesical or vesico#vagina* or cysto#vaginal)) or ((rectovaginalis or rectal vaginal or recto#vaginal or rectum vagina or bladder vaginal or vesicovaginalis or vagino#vesical or vesico#vagina*) N3 fistula) or VVF or RVF |
| *Oncology* | ((Cervi* or colli or endocervi*) ADJ5 (dysplasia or atypical or neoplas* or tumo?r or cancer or carcinoma or malignancy or microcarcinoma or pre?carcinoma or pre?cancer)) or ((dysplasia or atypical or neoplas* or tumo?r or cancer or carcinoma or malignancy or microcarcinoma or pre?carcinoma or pre?cancer) ADJ5 (Cervi* or colli or endocervi*)) or CIN or cervi* intraepithelial neoplasia or cervi* carcinoma in situ or cervi* squamous cell carcinoma or cervi* interstitial neoplasia | uterine cervix dysplasia/ or exp uterine cervix tumor/ | Uterine Cervical Neoplasms/ or exp Uterine Cervical Dysplasia/ or Cervical Intraepithelial Neoplasia | (MH "Cervix Neoplasms+") OR (MH "Cervix Dysplasia") OR (MH "Cervical Intraepithelial Neoplasia") | ((Cervi* or colli or endocervi*) N5 (dysplasia or atypical or neoplas* or tumo#r or cancer or carcinoma or malignancy or microcarcinoma or pre#carcinoma or pre#cancer)) or ((dysplasia or atypical or neoplas* or tumo#r or cancer or carcinoma or malignancy or microcarcinoma or pre#carcinoma or pre#cancer) N5 (Cervi* or colli or endocervi*)) or CIN or cervi* intraepithelial neoplasia or cervi* carcinoma in situ or cervi* squamous cell carcinoma or cervi* interstitial neoplasia |
| *Lymphoma* | Lymphoma* or adenolymphoma* or cystadenoma lymphomatosum or lymph node tumo?r or lymphocytic tumo?r or lymphoid malignanc* or lymphoid neoplas* | exp lymphoma/ | exp lymphoma/ | (MH "lymphoma+") | Lymphoma* or adenolymphoma* or cystadenoma lymphomatosum or lymph node tumo#r or lymphocytic tumo#r or lymphoid malignanc* or lymphoid neoplas* |
| *Leukemia* | Aleuk?emia or leuk?emia or hemoblastoma or leuc?emia or leukemogenesis | exp leukemia/ | exp leukemia/ | (MH "Leukemia+") | Aleuk#emia or leuk#emia or hemoblastoma or leuc#emia or leukemogenesis |
| *Melanoma* | Melanoma* or melanomatosis or melanocarcinoma or melanomalignoma or melanomalignoma or n?evocarcinoma or pigmentary cancer | exp melanoma/ | exp melanoma/ | (MH "melanoma+") | Melanoma* or melanomatosis or melanocarcinoma or melanomalignoma or melanomalignoma or n#evocarcinoma or pigmentary cancer |
| *Anorexia* | Anorexia | anorexia/ or anorexia nervosa/ | anorexia/ or anorexia nervosa/ | (MH "anorexia") or (MH "anorexia nervosa") | anorexia |
| *Bulimia* | Bulimia or hyperrexia | bulimia/ | Bulimia nervosa/ | (MH "Bulimia nervosa") | Bulimia or hyperrexia |
| *Rape* | Rape or sexual assault | Exp rape/ | Sex Offenses/ or rape/ | (MH "Rape") or MH("Sexual Abuse") | Rape or sexual assault |
| *Anxiety* | ((Adjustment or anxiety or panic or post?traumatic stress) adj3 Disorder*) or PTSD or (post?traumatic adj3 (stress or syndrome or psych* or neurosis)) or panic attack* or to#ophobia* | Exp Anxiety disorder/ | Exp Anxiety disorders/ | (MH "Anxiety disorders+") OR (MH "Anxiety+") | ((Adjustment or anxiety or panic or post#traumatic stress) N3 Disorder*) or PTSD or (post#traumatic N3 (stress or syndrome or psych* or neurosis)) or panic attack* or to#ophobia* |
| *Bipolar disorder* | ((Bipolar or manic depress* or manio?depressive or mano depressive) adj3 (disorder or disease or illness or psychosis or depression or reaction or syndrome)) or ((disorder or disease or illness or psychosis or depression or reaction or syndrome) adj3 (Bipolar or manic depress* or manio?depressive or mano depressive)) or manic depression or bipolar mania or cyclothymia or mixed mania and depression | Exp bipolar disorder/ | Exp "Bipolar and Related Disorders"/ | (MH "Bipolar Disorder+") | ((Bipolar or manic depress* or manio#depressive or mano depressive) N3 (disorder or disease or illness or psychosis or depression or reaction or syndrome)) or ((disorder or disease or illness or psychosis or depression or reaction or syndrome) N3 (Bipolar or manic depress* or manio#depressive or mano depressive)) or manic depression or bipolar mania or cyclothymia or mixed mania and depression |
| *Major depressive disorder/postpartum blues/postpartum depression* | ((post?partum or puerper* or post?natal or maternal or major or unipolar or clinical) ADJ2 depressi*) or (depressi* adj2 (post?partum or puerper* or post?natal or maternal or major or unipolar or clinical)) or unipolar disorder or ppd or post?partum blues or baby blues or post?partum baby blues | Puerperal depression/ or major depression/ | Depressive Disorder, Major/ or Depression, Postpartum/ | (MH "Depression+") | ((post#partum or puerper* or post#natal or maternal or major or unipolar or clinical) N2 depressi*) or (depressi* N2 (post#partum or puerper* or post#natal or maternal or major or unipolar or clinical)) or unipolar disorder or ppd or post#partum blues or baby blues or post#partum baby blues |
| *Psychosis/puerperal psychosis/schizophrenic* | Psychos#s or encephalopschychosis or psychotic* or schizophrenia or schizophrenic or schizophreniform or hebephrenia or negative syndrome or positive syndrome or dementia pr?ecox | Exp psychosis/ | exp psychotic Disorders/ or exp Schizophrenia/ | (MH " Psychotic Disorders+") | Psychos#s or encephalopschychosis or psychotic* or schizophrenia or schizophrenic or schizophreniform or hebephrenia or negative syndrome or positive syndrome or dementia pr#ecox |
| *Co-incidental* | | |  |  |  |
| *Intimate partner violence* | Partner violence or spouse abuse or partner abuse or wife abuse or IPV or domestic violence or battered wom#n or battered wife or battered wive or wife beating or wive beating | Exp partner violence/ or battered woman/ or domestic violence/ | Domestic Violence/ or exp Intimate Partner Violence/ or Battered Women/ | (MH "Domestic Violence+") OR (MH "Dating Violence") OR (MH "Battered Women") | Partner violence or spouse abuse or partner abuse or wife abuse or IPV or domestic violence or battered wom#n or battered wife or battered wive or wife beating or wive beating |
| *Falls* | Fall* or slip* | Falling/ | Accidental Falls/ | (MH "Accidental Falls") | Fall* or slip* |
| *Motor vehicle accident (transport accidents)* | ((vehicl* or transport* or traffic or road or streetcar or automobile or car or motorcar or motorcycle) and (accident or crash or collision)) | Exp accident/ OR fire/ | Accidents, Traffic/ | (MH "Accidents, Traffic") | ((vehicl* or transport* or traffic or road or streetcar or automobile or car or motorcar or motorcycle) and (accident or crash or collision)) |
| *Accidental exposure to smoke, fire, flames* | Burn* or ((smok* or flame* or fire) and accident*) |  | Accidents, Home/ or Accidents, Occupational/ or accidents/ or Fires/ | (MH "Accidents, Home") OR (MH "Accidents, Occupational+") OR (MH "Accidents") OR (MH “Fires+”) | Burn* or ((smok* or flame* or fire) and accident*) |
| *Accidental poisoning and exposure to noxious substance* | Accident* and (overdose or poisoning) |  |  |  | Accident* and (overdose or poisoning) |
| *Accidental drowning and submersion* | (Drowning or submersion) and accident* |  | Exp Drowning/ | (MH "Drowning+") | (Drowning or submersion) and accident* |
| *Contact with venomous animals and plants* | (accident* or contact*) and (Poisonous or toxic or venomous) and (organism* or species or animal* or plant*) | Exp toxic organism/ | Exp Venoms/ | (MH " Venoms+") | (accident* or contact*) and (Poisonous or toxic or venomous) and (organism* or species or animal* or plant*) |
| *Exposure to force of nature* | Storm* or hurricane* or tornado* or cyclon* or typhoon* or wind* or rain* or lightning | Exp weather/ | Exp weather/ | (MH "Weather+") | Storm* or hurricane* or tornado* or cyclon* or typhoon* or wind* or rain* or lightning |

14. Unsafe induced septic abortion – MEDLINE and Embase

| 1 | (exp Spontaneous Abortion/ OR spontaneous abortio*.mp) AND (exp induced abortion/ OR induced abortio*.mp.) |
| --- | --- |
| 2 | (exp Induced Abortion/ OR (Pregnan* adj3 Terminat*).mp. OR f?eticid*.mp. OR exp Curettage/ OR suction curettage.mp. OR post?abortion.mp. OR obstetrical extraction.mp. OR (aborti* NOT abortive).mp OR (dilatation adj2 (extract* OR curettage)).mp.) |
| 3 | (exp spontaneous abortion/ OR (spontan* adj2 abortio*) OR miscarriag*) NOT 1 |
| 4 | **2 NOT 3** |
| 5 | exp Morbidity/ OR exp Intensive Care/ OR exp hospitalization/ OR exp Postoperative Complications/ OR exp Coma/ OR sequel?e.mp. OR Near-miss.mp. OR intensive care.mp. OR (maternal adj2 morbidit*.mp.) OR samm.mp. OR hospitali#atio*.mp. OR complication*.mp. OR post?operative complication*.mp. OR adverse effect*.mp. OR adverse outcome*.mp. OR coma.mp. |
| 6 | exp Endotoxemia/ OR exp Toxemia/ OR exp Pelvic Inflammatory Disease/ OR exp Sepsis/ OR exp puerperal disorder/ OR exp Systemic Inflammatory Response Syndrome/ OR exp Septic Shock/ OR exp Septicemia/ OR exp Adnexitis/ OR exp Peritonitis/ OR exp Bacteremia/ OR exp Reproductive Tract Infections/ OR tox?emia.mp. OR Endotox?emia.mp. OR Oophoritis.mp. OR (pelvi* ADJ2 (infection* OR inflammat*)).mp. OR sepsis.mp. OR septic*.mp. OR septic shock.mp. OR septic?emia.mp. OR systemic inflammatory response syndrome.mp. OR blood poisoning.mp. OR endometritis.mp. OR parametritis.mp. OR Adnexitis.mp. OR peritonitis.mp. OR pelvic inflammatory disease.mp. OR bacter?emi*.mp. OR salpingitis.mp. OR salpingo-oophoritis.mp. OR reproductive tract infection*.mp. |
| 7 | exp Uterine Hemorrhage/ OR exp Hemorrhage/ OR exp Postoperative hemorrhage/ OR exp Afibrinogenemia OR exp Hemorrhagic Shock/ OR exp Anemia/ OR (uter* ADJ (bleeding or h?emorrhage)).mp. OR h?emorrhag*.mp. OR h?emorrhag* shock.mp. OR afibrinogen?emia.mp. OR defibrination syndrome.mp. OR intravascular coagulation.mp. OR (retained ADJ (product* OR placenta)).mp. OR blood tranfusion.mp. OR hypovol?emi*.mp. OR an?emia.mp. |
| 8 | exp Embolism OR exp Thromboembolism/ OR amniotic fluid embol*.mp. OR emboli*.mp. OR embolus.mp. OR pulmonary embol*.mp. OR thromboembol*.mp. |
| 9 | exp Shock/ OR exp Multiple Organ Failure/ OR Shock.mp. OR Multi-organ failure.mp. OR circulat* collapse.mp. OR multiple organ dysfunction syndrome.mp. OR mods.mp. |
| 10 | exp Renal Insufficiency/ OR exp Oliguria/ OR ((Kidney OR renal) ADJ (fail* OR insufficienc* OR tubular necrosis)).mp. OR oliguria.mp. |
| 11 | exp Uterine Rupture/ OR ((Uter* OR pelvi* OR cervi* OR genital*) AND (perforat* OR lacerat* OR tear* OR damage* OR trauma OR injur* OR rupture*)).mp. OR fistula*.mp. |
| 12 | exp Maternal Mortality/ OR maternal mortality.mp. OR ((exp Pregnancy Complications/ OR exp Pregnancy/ OR exp Mothers/ OR (pregnan* OR parturition OR maternal OR mother* OR childbirth).mp.) AND (exp Death/ OR exp Mortality/ OR death.mp. OR mortalit*.mp. OR fatalit*.mp.)) |
| 13 (“all morbidity”) | 5 OR 6 OR 7 OR 8 OR 9 OR 10 OR 11 OR 12 |
| Final search | **4 AND 13** |

Source: Adler AJ, Filippi V, Thomas SL, Ronsmans C. Quantifying the global burden of morbidity due to unsafe abortion: magnitude in hospital-based studies and methodological issues. *Int J Gynecol Obstet.* 2012; 118 Suppl 2:S65–77.

14. Unsafe induced septic abortion – CINAHL

| 1 | ((MH "Abortion, Spontaneous+") OR spontaneous abortio*) AND ((MH "Abortion, Induced+") OR induced abortio*) |
| --- | --- |
| 2 | ((MH "Abortion, Spontaneous+") OR (Pregnan* N3 Terminat*) OR f#eticid* OR (MH "Curettage+") OR suction curettage OR post#abortion OR obstetrical extraction OR (aborti* NOT abortive) OR (dilatation N2 (extract* OR curettage))) |
| 3 | ((MH "Abortion, Spontaneous+") OR (spontan* N2 abortio*) OR miscarriag*) NOT 1 |
| 4 | **2 NOT 3** |
| 5 | (MH "Morbidity+") OR (MH "Intensive Care+") OR (MH "hospitalization+") OR (MH "Postoperative Complications+") OR (MH "Coma") OR sequel#e OR Near-miss OR intensive care OR (maternal N2 morbidit*) OR samm OR hospitali?atio* OR complication* OR post#operative complication* OR adverse effect* OR adverse outcome* OR coma |
| 6 | (MH "Endotoxemia") OR (MH "Toxemia") OR (MH "Pelvic Inflammatory Disease+") OR (MH "Sepsis+") OR (MH "puerperal disorders+") OR (MH "Systemic Inflammatory Response Syndrome") OR (MH "Shock+") OR (MH "Peritonitis") OR (MH "Bacteremia") OR tox#emia OR Endotox#emia OR Oophoritis OR (pelvi* N2 (infection* OR inflammat*)) OR sepsis OR septic* OR septic shock OR septic#emia OR systemic inflammatory response syndrome OR blood poisoning OR endometritis OR parametritis OR Adnexitis OR peritonitis OR pelvic inflammatory disease OR bacter#emi* OR salpingitis OR salpingo-oophoritis OR reproductive tract infection* |
| 7 | (MH "Uterine Hemorrhage+") OR (MH "Hemorrhage+") OR (MH "Postoperative hemorrhage") OR (MH "Afibrinogenemia") OR (MH "Shock, Hemorrhagic") OR (MH "Anemia+") OR (uter* N (bleeding or h#emorrhage)) OR H#emorrhag* OR h#emorrhag* shock OR Afibrinogen#emia OR defibrination syndrome OR intravascular coagulation OR (retained N (product* OR placenta)) OR blood tranfusion OR hypovol#emi* OR an#emia |
| 8 | (MH "Embolism+") OR (MH "Thromboembolism+") OR amniotic fluid embol* OR emboli* OR embolus OR pulmonary embol* OR thromboembol* |
| 9 | (MH "Shock+") OR (MH "Multiple Organ Dysfunction Syndrome") OR Shock OR Multi-organ failure OR circulat* collapse OR multiple organ dysfunction syndrome OR mods |
| 10 | (MH "Renal Insufficiency+") OR (MH "Oliguria") OR ((Kidney OR renal) N (fail* OR insufficienc* OR tubular necrosis)) OR oliguria |
| 11 | (MH "Uterine Rupture") OR ((Uter* OR pelvi* OR cervi* OR genital*) AND (perforat* OR lacerat* OR tear* OR damage* OR trauma OR injur* OR rupture*)) OR fistula* |
| 12 | (MH "Maternal Mortality") OR maternal mortality OR (((MH "Pregnancy Complications+") OR (MH "Pregnancy+") OR (MH "Mothers+") OR (pregnan* OR parturition OR maternal OR mother* OR childbirth)) AND ((MH "Death+") OR (MH "Mortality+") OR death OR mortalit* OR fatalit*)) |
| 13 (“all morbidity”) | 5 OR 6 OR 7 OR 8 OR 9 OR 10 OR 11 OR 12 |
| Final search | **4 AND 13** |

Source: Adler AJ, Filippi V, Thomas SL, Ronsmans C. Quantifying the global burden of morbidity due to unsafe abortion: magnitude in hospital-based studies and methodological issues. *Int J Gynecol Obstet.* 2012; 118 Suppl 2:S65–77.
