# Supplementary material for: The frequency of maternal morbidity: A systematic review of systematic reviews
Source: Int J Gynaecol Obstet. 2018 May 23;141(Suppl Suppl 1):20–38. doi: 10.1002/ijgo.12468 (PMC6001670; doi:10.1002/ijgo.12468)
Supplement: Supplementary file 2 — Appendix S2. Quality assessment methods. [file IJGO-141-20-s002.docx]

**Appendix S2.** Quality assessment methods.

For the purpose of quality assessment, we adapted the quality assessment tool proposed by Mann et al. [1], which is a modified version of the Overview Quality Assessment Questionnaire (OQAQ). We excluded one question: Was the validity of all studies referred to in the text assessed using appropriate criteria (either in selecting study for inclusion or in analyzing the studies that are cited)? It was excluded as it was unclear how to use it in the context of our review and it did not seem to add value in the assessment of the studies included. We also added two questions: (1) Was the search strategy comprehensive? (i.e. exhaustive list of synonyms and all combinations of terms); and (2) Were the sources of the data reported? (i.e. explicit statement on whether each study includes population- or facility-based data). The first additional question was intended to capture the quality of the strategy used to identify studies, as we considered that none of the existing questions would capture that dimension. The second additional question was an attempt to further characterize the quality of the estimates used, since recruiting from facilities might affect the generalizability of the estimates, particularly in low- and middle-income countries, where health services coverage is not always universal. Furthermore, we decided to establish clearer criteria on the application of this quality assessment tool. Before its application, we discussed and decided which specific characteristics of the studies would result in specific answers to each question. Details of the application are provided in the table below. “Yes”, “Partially”, “No”, and “Unclear” answers were then color-coded using a traffic light system, using green, orange, red, and grey, respectively, please refer to Figure 2.

| Question | Application |
| --- | --- |
| 1. Were the search methods to find evidence (original search) on the primary question(s) stated? | - Yes if the methods were reported - Partially if some aspect of the methods was not reported - No if search methods were not stated |
| 1. Was the search strategy comprehensive? (i.e. exhaustive list of synonyms and all combinations of terms) | - Yes if a search strategy was provided and included not only search terms but also both a list of synonyms and all combinations of terms (or an explanation of how search terms were combined) - Partially if a search strategy was provided and included either a list of synonyms or an explanation of how search terms were combined - No if a search strategy was provided but there was no attempt to include synonyms for the search terms and there was no indication of how the search terms were combined - Unclear if the search strategy was not provided |
| 1. Was the search for evidence reasonably comprehensive? (i.e. statement that at least two electronic sources were searched plus supplemental searches; sources should be named) | - Yes if at least two databases were searched - No if only one database was searched - Unclear if no information was provided on the databases used |
| 1. Were the criteria used to decide which studies to include in the review reported? (i.e. explicit statement of inclusion/exclusion criteria) | - Yes if both inclusion and exclusion criteria were reported - Partially if only inclusion or exclusion criteria were reported - Unclear if no criteria were reported |
| 1. Was bias in the selection of studies avoided? (i.e. explicit statement of whether publication status or language of publication were used as exclusion criteria) | - Yes if grey literature was included and non-English publications were eligible - Partially if grey literature was included or non-English publications were eligible - No if grey literature and non-English publications were excluded - Unclear if no information was provided on grey literature or non-English publications |
| 1. Were the criteria used to assess the validity of the studies included reported? (i.e. explicit statement of the quality assessment tool used to assess the primary studies included) | - Yes if quality assessment was performed and the tool used was reported - Partially if quality assessment was performed but no information was provided on the tool used - No if explicitly mentioned that no quality assessment was performed - Unclear if no information was provided on quality assessment |
| 1. Was the data extraction process reported? (i.e. explicit statement on data extraction process, e.g. use of data extraction form, independent extractors, management of duplicates, consensus procedure for disagreements) | - Yes if at least double independent data extraction was performed and the procedure for disagreement was reported - Partially if at least double independent data extraction was performed or the procedure for disagreement was reported - No if single data extraction was performed - Unclear if no information was provided on data extraction |
| 1. Were the methods used to combine the findings of the relevant studies (to reach a conclusion) reported? (i.e. explicit statement of methods used to combine findings) | - Yes if methods to combine the findings were reported - Partially if methods were reported incompletely - No if methods were not reported |
| 1. Were the findings of the relevant studies combined appropriately relative to the primary question the review addresses? (i.e. explicit statement of whether or findings were (a) combined, and/or (b) combined using appropriate methods)  - If a summary estimate is reported in papers, but estimation method is omitted, answer “No”) | - Yes if methods to combine findings were reported as being appropriate - Partially if methods were reported incompletely - No if the methods used were reported as being inappropriate - Unclear if findings were not combined and no reason was given |
| 1. Were characteristics of the included studies described sufficiently? (e.g. in a table) | - Yes if there is detailed information for each individual study - Partially if individual studies are described but not in sufficient detail - No if characteristics of included studies are not described |
| 1. Were the sources of the data reported? (i.e. explicit statement on whether each study includes population- or facility-based data) | - Yes if source was reported - Partially if source of data was incomplete - No if no information was provided on the source of the data |
| 1. Was potential publication bias assessed? | • Yes if publication bias was assessed  • No if publication bias was not assessed |
| 1. Were the conclusions made by the authors supported by the data and/or analysis reported in the overview? | • Yes if all conclusions were supported  • Partially if some conclusions were supported but not all  • No if conclusions were not supported  • Unclear if no specific conclusions regarding the estimates were provided |

**Reference**

[1] Mann R, Gilbody S, Adamson J. Prevalence and incidence of postnatal depression: what can systematic reviews tell us? *Arch Womens Ment Health*. 2010;13:295–305.
