# Supplementary material for: The frequency of maternal morbidity: A systematic review of systematic reviews
Source: Int J Gynaecol Obstet. 2018 May 23;141(Suppl Suppl 1):20–38. doi: 10.1002/ijgo.12468 (PMC6001670; doi:10.1002/ijgo.12468)
Supplement: Supplementary file 3 — Appendix S3. List of available systematic reviews. [file IJGO-141-20-s003.docx]

**Appendix S3.** List of available systematic reviews.

***WHO MATERNAL MORBIDITY LIST [1]***

| **DIRECT MATERNAL MORBIDITY [eligible**  **systematic review found]** |
| --- |
| **Delivery/Termination** |
| Ectopic Pregnancy |
| Unsafe Induced Septic Abortion [2] |
| Retained Products of Conception [3] |
| Gestational Trophoblastic Disease |
| Obstructed Labor |
| **Hypertensive Disorders of Pregnancy** |
| Hypertensive Disorders (Chronic hypertension, Gestational  hypertension, Pre-eclampsia, HELLPS (hemolysis, elevated liver enzymes, low platelet count), Eclampsia) [4] |
| **Obstetric Hemorrhage** |
| Accreta/Increta/Percreta Placenta (Morbidly adherent placenta) |
| Placenta Previa [5] |
| Placental Abruption |
| Postpartum Hemorrhage [6,7] |
| **Pregnancy related Infection** |
| Mastitis/Breast Abscess |
| Chorioamnionitis |
| Puerperal Sepsis |
| UTI/Pyelonephritis |
| Tetanus |
| **Other Obstetric Complications** |
| **Embolic/Thrombotic** |
| Deep Vein Thrombosis [8,9] |
| Thrombophlebitis |
| Septic Ovarian/Pelvic Vein Thrombophlebitis |
| Amniotic Fluid Embolism^a^ [10,11] |
| **Gastrointestinal** |
| Nausea and Vomiting of Pregnancy [12] |
| Cholestasis of Pregnancy |
| **Cardiovascular** |
| Peripartum Cardiomyopathy |
| Mirror Syndrome |
| **Endocrine** |
| Diabetes Mellitus (Gestational Diabetes) [13–20] |
| Postpartum Thyroiditis |
| **Other** |
| Intentional Self Harm [21] |
| **Unanticipated Complications** |
| **Complications related to Anesthesia** |
| Aspiration Pneumonitis |
| Cerebral Anoxia |
| **Complications of Management** |
| *Spontaneous Vaginal Delivery* |
| Perineal Tear (3rd or 4th degree) [22] |
| *Episiotomy* |
| Episiotomy infection |
| *Instrumental Delivery* |
| Vaginal wall/perineal laceration |
| Urethral tear/damage |
| Vulval hematoma |
| *Cesarean delivery* |
| Uterine perforation |
| Postpartum Inversion of Uterus |
| Cesarean delivery Wound Infection |
| Postoperative ileus/ bowel obstruction |
| *Other* |
| Ovarian hyperstimulation syndrome |
| Nosocomial or hospital acquired infection (UTI, C diff, pneumonia) |
| **CO-INCIDENTAL** |
| **External injury in pregnancy** |
| Motor Vehicle Accident (Transport accidents) [21] |
| Accidental exposure to smoke, fire, flames [21] |
| Accidental poisoning and exposure to noxious substance [21] |
| Accidental drowning and submersion |
| Contact with venomous animals and plants |
| Exposure to force of nature |
| **Trauma in pregnancy** |
| Falls [21] |
| Intimate Partner Violence [23–26] |
| Rape |

^a^ Condition was added after consultation with authors of the WHO morbidity list following their suggestion [1].

| **INDIRECT MATERNAL MORBIDITY – CONDITIONS [eligible systematic review found]** |
| --- |
| Pre-existing Hypertension |
| Pre-exiting Diabetes Mellitus [17] |
| **Maternal infectious and parasitic diseases classified elsewhere but complicating pregnancy, childbirth and the puerperium** |
| HIV/AIDS [27] |
| Tuberculosis Mycobacterium |
| Malaria [28,29] |
| *Sexually Transmitted Infections* |
| Chlamydia [28,30] |
| Anogenital warts [31] |
| Herpes Simplex |
| Syphilis [28,30] |
| *Other* |
| Candidiasis |
| Influenza |
| Pneumonia |
| Infectious Hepatitis (A, B, C, E) [32–35] |
| Varicella Zoster |
| Cholera |
| **Other Maternal Diseases Classifiable Elsewhere but Complicating Pregnancy, Childbirth and the Puerperium** |
| *Acquired Anemia* |
| Anemia due to vitamin B12 and/or folate deficiency |
| Iron Deficiency Anemia |
| *Hereditary Anemia* |
| Sickle Cell Anemia |
| Thalassemia |
| **Other diseases in the blood and blood forming organs and certain disorders involving the immune mechanism complicating pregnancy, childbirth and the puerperium** |
| Idiopathic Immune Thrombocytopenia (ITP) |
| **Endocrine, nutritional and metabolic diseases complicating pregnancy, childbirth and the puerperium** |
| Hyperparathyroidism |
| *Thyroid disorders* |
| Hyperthyroidism |
| Hypothyroidism |
| **Mental disorders and diseases of the nervous system complicating pregnancy, childbirth and the puerperium** |
| *Anxiety Disorders* |
| Adjustment Disorder |
| Anxiety Disorder [36–38] |
| Panic Disorder [36,37] |
| Post-traumatic Stress Disorder [38,39] |
| Tocophobia (specific isolated phobias) |
| *Mood Disorders* |
| Bipolar Disorder [40] |
| Major Depressive Disorder [41] |
| Postpartum Blues [42] |
| Postpartum Depression [38,41–43] |
| Psychosis |
| Puerperal Psychosis |
| Schizophrenia |
| **Diseases of the circulatory system complicating pregnancy, childbirth and the puerperium** |
| Acquired and Congenital Structural Heart Disease (including valvular heart disease) |
| Aortic Dissection |
| Arrhythmia |
| Cardiomyopathy (dilated and restrictive) |
| **Diseases of the respiratory system complicating pregnancy, childbirth and the puerperium** |
| Asthma |
| Obstructive Sleep Apnea |
| Pulmonary Embolism [8,9] |
| **Diseases of the digestive system complicating pregnancy, childbirth and the puerperium** |
| Anal Fissure |
| Hemorrhoids |
| Cholecystitis/cholylethiasis |
| Gastrointestinal Esophageal Reflux Disease (GERD) |
| Inflammatory Bowel Disease |
| **Diseases of the Genitourinary System** |
| Acute and Chronic Kidney Disease |
| **Diseases of the skin and subcutaneous tissue complicating pregnancy, childbirth and the puerperium** |
| *Pregnancy Specific Dermatoses* |
| Eczema (Atopic dermatitis) |
| Prurigo of Pregnancy (Diseases of the skin and subcutaneous tissue complicating pregnancy, childbirth and the puerperium) |
| Pruritic Urticarial Papules and Plaques of Pregnancy (PUPP) or Polymorphic Eruption of Pregnancy (PEP) Linea nigra |
| *Dermatoses aggravated by pregnancy* |
| Acne |
| Psoriasis |
| **Other specified diseases and conditions complicating pregnancy, childbirth and the puerperium** |
| **Diseases of the Nervous System** |
| Bell's Palsy |
| Carpal Tunnel [44] |
| Migraine |
| Multiple Sclerosis |
| Restless Leg Syndrome |
| Seizure disorder (excluding eclampsia) |
| **Diseases of the Musculoskeletal System and Connective Tissue** |
| Inflammatory arthritis |
| Ankylosing spondylitis |
| Rheumatoid Arthritis |
| Systemic Lupus Erythematosus (SLE) |
| Non-inflammatory arthritis |
| Back pain |
|  |
| Incontinence (urge, stress) [45–47] |
| Uterine/Uterovaginal Prolapse (including cystocele) |
| Rectovaginal fistula [48–50] |
| Vesicovaginal fistula [48–50] |
| **Oncology** |
| Cervical Dysplasia/Neoplasia |
| Lymphoma |
| Leukemia |
| Melanoma |
| **Nutritional** |
| Anorexia Nervosa |
| Bulimia Nervosa |

**Key**

|  | Available |
| --- | --- |
|  | Available but no estimates extracted |
|  | Blank – not available |

**References**

1. Chou D, Tunçalp Ö, Firoz T, et al. Constructing maternal morbidity – towards a standard tool to measure and monitor maternal health beyond mortality. *BMC Pregnancy Childbirth*. 2016;16:45.
2. Adler AJ, Filippi V, Thomas SL, Ronsmans C. Incidence of severe acute maternal morbidity associated with abortion: a systematic review. *Trop Med Int Health*. 2012;17:177–90.
3. Cheung WMC, Hawkes A, Ibish S, Weeks AD. The retained placenta: historical and geographical rate variations. *J Obstet Gynaecol.* 2011;31:37–42.
4. Abalos E, Cuesta C, Carroli G, et al. Pre-eclampsia, eclampsia and adverse maternal and perinatal outcomes: a secondary analysis of the World Health Organization Multicountry Survey on Maternal and Newborn Health. *BJOG.* 2014;121:14–24.
5. Cresswell JA, Ronsmans C, Calvert C, Filippi V. Prevalence of placenta praevia by world region: a systematic review and meta-analysis. *Trop Med Int Health.* 2013;18:712–24.
6. Carroli G, Cuesta C, Abalos E, Gulmezoglu AM. Epidemiology of postpartum haemorrhage: a systematic review. *Best Pract Res Clin Obstet Gynaecol*. 2008;22:999–1012.
7. Calvert C, Thomas SL, Ronsmans C, Wagner KS, Adler AJ, Filippi V. Identifying regional variation in the prevalence of postpartum haemorrhage: a systematic review and meta-analysis. *PLoS One*. 2012;7:e41114.
8. Meng K, Hu X, Peng X, Zhang Z. Incidence of venous thromboembolism during pregnancy and the puerperium: a systematic review and meta-analysis. *J Matern Fetal Neonatal Med.* 2015;28:245–53.
9. Kourlaba G, Relakis J, Kontodimas S, Holm MV, Maniadakis N. A systematic review and meta-analysis of the epidemiology and burden of venous thromboembolism among pregnant women. *Int J Gynecol Obstet.* 2016;132:4–10.
10. Conde-Agudelo A, Romero R. Amniotic fluid embolism: an evidence-based review. *Am J Obstet Gynecol*. 2009;201:445.e1–13.
11. Frati P, Foldes-Papp Z, Zaami S, Busardo FP. Amniotic fluid embolism: what level of scientific evidence can be drawn? A systematic review. *Curr Pharm Biotechnol.* 2014;14:1157–62.
12. Einarson TR, Piwko C, Koren G. Quantifying the global rates of nausea and vomiting of pregnancy: a meta analysis. *J Popul Ther Clin Pharmacol.* 2013;20:e171–83.
13. Buckley BS, Harreiter J, Damm P, et al. Gestational diabetes mellitus in Europe: prevalence, current screening practice and barriers to screening. A review. *Diabet Med J Br Diabet Assoc.* 2012;29:844–54.
14. Hunt KJ, Schuller KL. The increasing prevalence of diabetes in pregnancy. *Obstet Gynecol Clin North Am*. 2007;34:173–99.
15. Macaulay S, Dunger DB, Norris SA. Gestational diabetes mellitus in Africa: a systematic review. *PLoS One*. 2014;9:e97871.
16. Mwanri AW, Kinabo J, Ramaiya K, Feskens EJ. Gestational diabetes mellitus in sub-Saharan Africa: systematic review and metaregression on prevalence and risk factors. *Trop Med Int Health*. 2015;20:983–1002.
17. Kanguru L, Bezawada N, Hussein J, Bell J. The burden of diabetes mellitus during pregnancy in low- and middle-income countries: a systematic review. *Glob Health Action*. 2014;7.
18. Schneider S, Bock C, Wetzel M, Maul H, Loerbroks A. The prevalence of gestational diabetes in advanced economies. *J Perinat Med*. 2012;40:511–20.
19. Zhu Y, Zhang C. Prevalence of gestational diabetes and risk of progression to Type 2 diabetes: a global perspective. *Curr Diab Rep*. 2016;16:7.
20. Hirst JE, Raynes-Greenow CH, Jeffery HE. A systematic review of trends of gestational diabetes mellitus in Asia. *J Diabetol*. 2012;3:5.
21. Mendez-Figueroa H, Dahlke JD, Vrees RA, Rouse DJ. Trauma in pregnancy: an updated systematic review. *Am J Obstet Gynecol.* 2013;209:1–10.
22. Villot A, Deffieux X, Demoulin G, Rivain A-L, Trichot C, Thubert T. Management of third and fourth degree perineal tears: a systematic review [in French]. *J Gynecol Obstet Biol Reprod (Paris).* 2015;44:802–11.
23. Han A, Stewart DE. Maternal and fetal outcomes of intimate partner violence associated with pregnancy in the Latin American and Caribbean region. *Int J Gynecol Obstet.* 2014;124:6–11.
24. Shamu S, Abrahams N, Temmerman M, Musekiwa A, Zarowsky C. A systematic review of African studies on intimate partner violence against pregnant women: prevalence and risk factors. *PLoS One*. 2011;6:e17591.
25. Puccia MI, Mamede MV. Integrative review regarding intimate partner violence in pregnancy [in Portuguese]. *Rev Eletr Enf*. 2012;14:944–56.
26. Liepe K, Blättner B. Violence during pregnancy: prevalence studies in OECD countries [in German]. *Gesundheitswesen*. 2013;75:473–480.
27. Drake AL, Wagner A, Richardson B, John-Stewart G. Incident HIV during pregnancy and postpartum and risk of mother-to-child HIV transmission: a systematic review and meta-analysis. *PLoS Med*. 2014;11:e1001608.
28. Chico RM, Mayaud P, Ariti C, Mabey D, Ronsmans C, Chandramohan D. Prevalence of malaria and sexually transmitted and reproductive tract infections in pregnancy in sub-Saharan Africa: a systematic review. *JAMA*. 2012;307:2079–86.
29. Roberts T, Gravett CA, Velu PP, et al. Epidemiology and aetiology of maternal parasitic infections in low- and middle-income countries. *J Glob Health*. 2011;1:189–200.
30. Joseph Davey DL, Shull HI, Billings JD, Wang D, Adachi K, Klausner JD. Prevalence of curable sexually transmitted infections in pregnant women in low- and middle-income countries from 2010 to 2015: a systematic review. *Sex Transm Dis*. 2016;43:450–8.
31. Banura C, Mirembe FM, Orem J, Mbonye AK, Kasasa S, Mbidde EK. Prevalence, incidence and risk factors for anogenital warts in Sub Saharan Africa: a systematic review and meta analysis. *Infect Agent Cancer*. 2013;8:1.
32. Merrill RM, Hunter BD. Seroprevalence of markers for hepatitis B viral infection. *Int J Infect Dis.* 2011;15:e78–121.
33. Mora N, Adams WH, Kliethermes S, et al. A synthesis of hepatitis C prevalence estimates in Sub-Saharan Africa: 2000–2013. *BMC Infect Dis*. 2016;16:283.
34. Rao VB, Johari N, du Cros P, Messina J, Ford N, Cooke GS. Hepatitis C seroprevalence and HIV co-infection in sub-Saharan Africa: a systematic review and meta-analysis. *Lancet Infect Dis*. 2015;15:819–24.
35. Riou J, Aït Ahmed M, Blake A, et al. Hepatitis C virus seroprevalence in adults in Africa: a systematic review and meta-analysis. *J Viral Hepat*. 2016;23:244–55.
36. Goodman JH, Chenausky KL, Freeman MP. Anxiety disorders during pregnancy: a systematic review. *J Clin Psychiatry*. 2014;75:e1153–84.
37. Goodman JH, Watson GR, Stubbs B. Anxiety disorders in postpartum women: a systematic review and meta-analysis. *J Affect Disord.* 2016;203:292–331.
38. Sawyer A, Ayers S, Smith H. Pre- and postnatal psychological wellbeing in Africa: a systematic review. *J Affect Disord*. 2010;123:17–29.
39. Grekin R, O’Hara MW. Prevalence and risk factors of postpartum posttraumatic stress disorder: a meta-analysis. *Clin Psychol Rev.* 2014;34:389–401.
40. Sharma V, Pope CJ. Pregnancy and bipolar disorder: a systematic review. *J Clin Psychiatry*. 2012;73:1447–55.
41. Norhayati MN, Hazlina NH, Asrenee AR, Emilin WM. Magnitude and risk factors for postpartum symptoms: a literature review. *J Affect Disord*. 2015;175:34–52.
42. Schmied V, Johnson M, Naidoo N, et al. Maternal mental health in Australia and New Zealand: a review of longitudinal studies. *Women Birth J Aust Coll Midwives.* 2013;26:167–78.
43. Parsons C, Young K, Rochat T, Kringelbach M, Stein A. Postnatal depression and its effects on child development: a review of evidence from low- and middle-income countries. *Br Med Bull*. 2012;101:57–79.
44. Padua L, Di Pasquale A, Pazzaglia C, Liotta GA, Librante A, Mondelli M. Systematic review of pregnancy-related carpal tunnel syndrome. *Muscle Nerve*. 2010;42:697–702.
45. Cerruto MA, D’Elia C, Aloisi A, Fabrello M, Artibani W. Prevalence, incidence and obstetric factors’ impact on female urinary incontinence in Europe: a systematic review. *Urol Int*. 2013;90:1–9.
46. Sangsawang B, Sangsawang N. Stress urinary incontinence in pregnant women: a review of prevalence, pathophysiology, and treatment. *Int Urogynecology J*. 2013;24:901–12.
47. Thom DH, Rortveit G. Prevalence of postpartum urinary incontinence: a systematic review. *Acta Obstet Gynecol Scand*. 2010;89:1511–22.
48. Adler AJ, Ronsmans C, Calvert C, Filippi V. Estimating the prevalence of obstetric fistula: a systematic review and meta-analysis. *BMC Pregnancy Childbirth*. 2013;13:1–14.
49. Cowgill KD, Bishop J, Norgaard AK, Rubens CE, Gravett MG. Obstetric fistula in low-resource countries: an under-valued and under-studied problem – systematic review of its incidence, prevalence, and association with stillbirth. *BMC Pregnancy Childbirth*. 2015;15:193.
50. Zheng AX, Anderson FW. Obstetric fistula in low-income countries. *Int J Gynecol Obstet*. 2009;104:85–9.
