# Supplementary material for: The frequency of maternal morbidity: A systematic review of systematic reviews
Source: Int J Gynaecol Obstet. 2018 May 23;141(Suppl Suppl 1):20–38. doi: 10.1002/ijgo.12468 (PMC6001670; doi:10.1002/ijgo.12468)
Supplement: Supplementary file 4 — Appendix S4. Details of direct and indirect morbidity estimates. [file IJGO-141-20-s004.docx]

**Appendix S4.** Details of direct and indirect morbidity estimates.

**Table 1.** Details of direct morbidity estimates.

| Author | Type of estimate | Upper | Lower | Point estimate | List of countries (the extracted estimate may not include/reflect data from all listed countries) | Assessment method details | Notes on estimates | Sample size of included studies (total or range) |
| --- | --- | --- | --- | --- | --- | --- | --- | --- |
| Adler, 2012 | Median | 5.30% | 0.435% | 0.60% | Cambodia, Iran, Ethiopia, Kenya, Burkina Faso, South Africa, Egypt, Zimbabwe | Based on clinical diagnoses representing organ failure or dysfunction, including vascular dysfunction, immunological dysfunction or other organ dysfunction. Diagnoses included peritonitis, tetanus, gangrenous uterus, severe trauma with uterine perforation, gut injury, or bowel injury. Studies only reporting broad and undefined clinical entities (such as “infection” or “hemorrhage”) and studies that relied on self-reported complications were excluded. |  | 6,870 – 2,964,323 |
| Einarson, 2013 | Weighted average rate (used inverse variance weighting, giving greater weight to larger studies as well as incorporating between-study variance) | 72.30% | 66.50% | 69.40% | Australia, India, United States of America (USA), Hong Kong, Taiwan, United Kingdom (UK), Canada, Sweden, Nigeria, Israel, Japan, Netherlands, South Africa |  | Estimates are for early pregnancy. | 93,753 |
| Einarson, 2013 | Weighted average rate (used inverse variance weighting, giving greater weight to larger studies as well as incorporating between-study variance) | 3.60% | 0.20% | 1.20% | Australia, USA, Sweden, Hong Kong, Canada, Scotland, Japan, Norway |  |  | 4,977,340 |
| Buckley, 2012 | Range | 22.30% | 0.70% |  | Norway, Finland, Sweden, Latvia, UK, Ireland, Denmark, Lithuania, Germany, Poland, France, Belgium, Czech Republic, Austria, Switzerland, Hungary, Slovenia, Spain, Portugal, Italy | Diagnostic criteria included World Health Organization (WHO), American Diabetes Association (ADA), National Diabetes Data Group (NDDG), O'Sullivan, and Carpenter and Coustan, as well as unknown criteria (1 study). | Population source: Most studies are population-based, a few are from tertiary hospitals; the authors said they excluded studies with clear risk of selection bias and high-risk populations. | 179 – 292,170 |
| Hirst, 2012 | Range | 17.70% | 0.56% |  | China, Hong Kong, Japan, Korea, Thailand, India, Pakistan | Diagnostic criteria included WHO, Japan Society of Obstetrics & Gynaecology, NDDG, O'Sullivan, and Carpenter and Coustan. | Only includes studies with similar methods within each country to compare trends. LMICs included have universal screening, hence we consider it an attempt to achieve population-based data. | 157,799 |
| Hunt, 2007 | Range | 22.30% | 1.20% |  | USA, Canada, Brazil, UK, Netherlands, Sweden, Denmark, Italy, Turkey, Iran, Bahrain, Ethiopia, India, Australia, China, Japan | Diagnostic criteria included WHO, NDDG, Carpenter and Coustan, ADA. Information on this is missing for several studies. Includes 1 study with self-report diagnosis. |  | 741 – 329,671 |
| Kanguru, 2014 | Range | 17.25% | 0.40% |  | India, Sri Lanka, Bangladesh, China, Ethiopia, Argentina, Brazil, Cuba |  |  | 172 – 105,473 |
| Macaulay, 2014 | Range | 13.90% | 0.00% |  | Ethiopia, Morocco, Mozambique, Nigeria, South Africa, Tanzania | Diagnostic criteria included WHO, Carpenter and Coustan, NDDG, institutional protocols based on fast blood glucose, and others. |  | 109 – 12,030 |
| Mwanri, 2015 | Weighted mean (random effect model) | 10.01% | 1.68% | 5.06% | **Nigeria, South Africa, Tanzania, Cameroon, Congo | Diagnostic criteria included WHO, ADA, but also institutional ones, fasting blood glucose, etc. | Countries list and sample size is based on a table with all primary studies (published after 2000) included in the systematic reviews. 4 of the studies (all published after 2000) had a representative sample of women (from quality assessment of the authors). Hence it might represent the ones included in the extracted estimate presented here. | 189 – 12,030** |
| Schneider, 2012 | Range | 11.60% | 1.70% |  | USA, Canada, Australia, Spain, Italy, UK, Denmark, Finland, Sweden, Germany | Self-report/insulin test/glucose therapy/clinical diagnosis. Diagnostic criteria included NDDG, Diabetic Pregnancy Study Group (DPSG), WHO, Canadian Diabetes Association (CDA), ADA, or other without further specification. | Population sources: Clinical population and birth registry. | 1450 – 58,922,266 |
| Zhu, 2016 | Median | 22.30% | 1.80% | 5.80% | Norway, UK, Belgium, Hungary, Spain, France, Turkey, Switzerland, Greenland, Germany, Sweden, Ireland | Diagnostic criteria included WHO, NDDG, International Association of the Diabetes and Pregnancy Study Groups (IADPSG), Carpenter and Coustan, and other not specified. |  | Not clear |
| Zhu, 2016 | Median | 11.90% | 6.50% | 7.00% | Barbados, USA, Trinidad and Tobago, Canada | Diagnostic criteria included WHO, NDDG, IADPSG, Carpenter and Coustan, and other not specified. |  | Not clear |
| Zhu, 2016 | Median | 16.60% | 7.10% | 11.20% | Cuba, Brazil | Diagnostic criteria included WHO, IADPSG, Carpenter and Coustan, and other not specified. |  | Not clear |
| Zhu, 2016 | Median | 24.50% | 8.40% | 12.90% | United Arab Emirates (UAE), Qatar, Bahrain, Israel, Iran |  |  | Not clear |
| Zhu, 2016 | Median | 9.50% | 8.20% | 8.90% | Nigeria, Tanzania |  |  | Not clear |
| Zhu, 2016 | Median | 18.30% | 8.10% | 11.70% | Malaysia, India, Bangladesh, Sri Lanka | Diagnostic criteria included WHO, NDDG, IADPSG, Carpenter and Coustan, and other not specified. |  | Not clear |
| Zhu, 2016 | Median | 25.10% | 4.50% | 11.70% | Singapore, Thailand, Vietnam, China, New Zealand, Australia, Japan | Diagnostic criteria included WHO, NDDG, IADSPG, Carpenter and Coustan, and other not specified. |  | Not clear |
| Cresswell, 2013 | Weighted mean (random effect model) | 0.59% | 0.45% | 0.52% | Bosnia & Herzegovina, Australia, Democratic Republic of the Congo (DRC), Congo, Croatia, Cuba, Finland, Greece, Italy, Iran, Israel, Japan, Jordan, Korea, Norway, Oman, Saudi Arabia, Serbia, Singapore, Spain, Taiwan, Turkey, UAE, UK, USA | Confirmed at ultrasound prior to delivery, or confirmed at delivery; not reported for 17 studies. | Countries list and sample size is for all the primary studies included in the systematic review, not just the ones underlining the extracted estimate.  Facility-based studies were excluded if local or national (if local data unavailable) skilled birth attendance rate was <95%. | 21,749,944 |
| Cheung, 2012 | Median | 6.26% | 2.00% | 2.67% | USA, UK, Australia, Netherlands |  | Population source: Only 3 studies used both home and hospital deliveries; insufficient details to assess whether these studies are underlying the extracted estimate presented here. | 1,000 – 45,852 |
| Cheung, 2012 | Median | 4.60% | 1.05% | 1.55% | Vietnam, Shanghai, Hong Kong |  | Population source: Only 3 studies used both home and hospital deliveries; insufficient details to assess whether these studies are underlying the extracted estimate presented here. | 1,032 – 2,350 |
| Cheung, 2011 | Median | 5.42% | 0.60% | 2.40% | USA, UK, Australia, Norway, Sweden, Netherlands |  | Population source: Only 3 studies used both home and hospital deliveries; insufficient details to assess whether these studies are underlying the extracted estimate presented here. | 1,000 – 19,2321 |
| Cheung, 2011 | Median | 0.57% | 0.008% | 0.43% | Vietnam, Nigeria, Saudi Arabia, India |  | Population source: Only 3 studies used both home and hospital deliveries; insufficient details to assess whether these studies are underlying the extracted estimate presented here. | 1,235 – 26,315 |
| Abalos, 2013 | Mean (range) | 4.20% | 1.20% | 2.30% | Benin, Côte d'Ivoire, Nigeria, South Africa, Argentina, Brazil, Canada, Chile, Mexico, USA, Kuwait, Morocco, Denmark, France, Greece, Israel, Netherlands, Norway, Portugal, Slovakia, Spain, Turkey, UK, India, Indonesia, Thailand, Australia, Japan, Korea, New Zealand, Singapore |  | Population source: Only 9.6% of the data sets reporting pre-eclampsia and 9.5% of those reporting eclampsia showed national data. | 37,652,006 |
| Abalos, 2013 | Mean (range) | 2.70% | 0.10% | 1.10% | Benin, Côte d'Ivoire, Nigeria, South Africa, Argentina, Brazil, Chile, Mexico, USA, Afghanistan, Iran, Kuwait, Morocco, Pakistan, Saudi Arabia, Syrian Arab Republic, Georgia, Ireland, Netherlands, Portugal, Spain, Turkey, UK, India, Indonesia, Thailand, Australia, Singapore |  | Population source: Only 9.6% of the data sets reporting pre-eclampsia and 9.5% of those reporting eclampsia showed national data. | 38,006,992 |
| Calvert, 2012 | Weighted mean (random effect model) | 12.10% | 9.60% | 10.80% | Gambia, Tanzania, Guinea Bissau, Argentina, Uruguay, Barbados, Cuba, Jamaica, Canada, USA, Hong Kong, India, Japan, Jordan, China, Singapore, Saudi Arabia, Thailand, UAE, Austria, Belgium, Norway, UK, France, Netherlands, Sweden, Switzerland, Australia, New Zealand | Objective, subjective, and unknown (21/104 data sets) methods of blood loss measurement were included. Objective methods yielded higher prevalence estimates. | Facility-based studies were excluded if local or national (if local data unavailable) skilled birth attendance rate was <95%. | 1,003,694 |
| Carroli, 2008 | Weighted mean (weighting the sample size of individual studies) | 6.05% | 6.00% | 6.02% | Not clear | Objective, subjective, and unspecified (14/55 studies) methods of blood loss measurement were included. Objective methods yielded higher prevalence. |  | 3,620,663 |
| Calvert, 2012 | Weighted mean (random effect model) | 3.20% | 2.40% | 2.80% | Gambia, Guinea Bissau, Argentina, Uruguay, Cuba, Canada, USA, Hong Kong, Japan, Jordan, China, Singapore, Thailand, UAE, Saudi Arabia, Vietnam, Belgium, UK, France, Netherlands, Norway, Sweden, Switzerland, Australia, Germany, Ireland, Spain | Objective, subjective, and unknown (6/69 datasets) methods of blood loss measurement were included. Objective methods yielded higher prevalence estimates. | Facility-based studies were excluded if local or national (if local data unavailable) skilled birth attendance rate was <95%. | 503,046 |
| Carroli, 2008 | Weighted mean (weighting the sample size of individual studies) | 1.71% | 1.64% | 1.67% | Not clear |  |  | 73,973 |
| Villot, 2015 | Range | 9.70% | 2.95% | 6.3250% | Not clear | Not clear |  | Not clear |
| Conde-Agudelo, 2009 | Weighted mean | 0.0072% | 0.006% | 0.0066% | USA, Canada |  |  | 49,917** |
| Conde-Agudelo, 2009 | Weighted mean | 0.0021% | 0.0017% | 0.0019% | UK, Finland, Sweden |  |  | 163,806** |
| Frati, 2013 | Mean (range) | 0.02% | 0.00% | 0.01% | Not clear | Clinical assessment |  | 23,757,610 |
| Kourlaba, 2015 | Weighted mean (random effect model) | 0.11% | 0.10% | 0.11% | **UK, Norway, Sudan, Canada, USA, Saudi Arabia, Australia | No information, but assessment methods appropriateness was part of the authors quality criteria. | Population source: From the titles of the individual papers, it appears that both population-based and hospital-based studies are included. Recruitment was part of the authors quality assessment. | 6,987 – 80,798,000** |
| Meng, 2015 | Weighted mean (random effect model) | 1.30% | 1.00% | 1.10% | China, USA, Sudan, Canada, Norway, Australia, Saudi Arabia, Belgium, Denmark, Sweden | Based on clinical assessment based on several internationally recognized clinical diagnostic criteria, e.g. International Classification of Diseases (ICD-9, ICD-10), or objective diagnostic criteria, e.g. ultrasound. |  | 13,683,000 |

**Table 2.** Details of indirect morbidity estimates.

| Author | Type of estimate | Upper | Lower | Point estimate | Countries list (** might not represented extracted estimate) | Assessment method detail | Notes | Sample size of included studies (total or range) |
| --- | --- | --- | --- | --- | --- | --- | --- | --- |
| Cerruto, 2013 | Range | 58.10% | 6.70% |  | Not clear | Questionnaires, some of which are validated, e.g. International Consultation on Incontinence Questionnaire – Short Form (ICIQ-SF). At least 1 study did not have clear information on assessment method. | Countries list for specific estimate is not clear but the following were definitely included: Sweden, Norway, and Spain.  Some recruitment happened within the gynecological departments. | 195 – 43,279 |
| Sangsawang, 2012 | Range | 75.00% | 26.00% |  | Norway, Germany, USA, Australia | Throughout the systematic review, they have included papers using self-report, validated questionnaires, interviews, and other more rigorous methods like pad test (detail not relevant to the estimate level). |  | 113 – 722 |
| Padua, 2010 | Range | 43.00% | 7.00% |  | Not clear | Neurophysiologically confirmed – standard sensory and motor nerve conduction studies. More sensitive tests in 3/5 studies. |  | 58 – 259 |
| Kanguru, 2014 | Range | 0.70% | 0.00% |  | Pakistan, India, Thailand, South Africa, Nigeria, Cuba |  |  | 950 – 12,030 |
| Liepe, 2013 | Range | 31.70% | 1.80% |  | Denmark, Turkey, Mexico, USA, Australia, Belgium, Japan | Publications not using validated questionnaires excluded. | Population source: At least 4 studies with unclear recruitment strategy. | Not clear |
| Puccia, 2012 | Range | 94.00% | 3.40% |  | UAE, Brazil, Mexico, Belgium, China, Nicaragua, Guatemala, Canada, USA | 5 studies not reported, 1 self-reported, remainder used validated scales or questionnaires designed by the authors. |  | Not clear |
| Shamu, 2011 | Weighted mean (fixed-effect meta-analysis) | 16.08% | 14.38% | 15.23% | Nigeria, South Africa, Zimbabwe, Uganda | Studies used both their ‘‘own’’ questionnaires, and employed commonly used and validated instruments, such as the Conflict Tactics Scale 2 (CTS2), Abuse Assessment Screen (AAS)], WHO questionnaires. |  | 178 – 652 |
| Sawyer, 2010 | Weighted mean (aggregate mean, weighted by the number of subjects in each of the contributing studies) | 9.50% | 13.10% | 11.30% | Nigeria, Morocco, Gambia | Measures used to screen for disorders varied between studies. The majority of studies conducted structured clinical interviews (n=20), 10 studies used self-administered measures, and 3 studies used both. 5 different questionnaire measures of depression were used: the Edinburgh Postnatal Depression Scale (EPDS) (Cox et al., 1987), Hospital Anxiety and Depression Scale (HADS; Zigmond and Snaith, 1983), Beck Depression Inventory (BDI; Beck et al., 1961), Self Rating Depression Scale (SRDS, Zung, 1965), and the Pitt Depression Questionnaire (PDQ; Pitt, 1968). These questionnaires examine depressed mood so are not diagnostic measures. However, with the exception of the PDQ, all have been validated against clinical interviews with African samples and have recommended cut-offs for probable depressive disorder. |  | 1,217 |
| Schmied, 2013 | Range | 20.50% | 8.70% |  | Australia, New Zealand |  | Cohorts were recruited both from general population and from antenatal care (ANC) clinics, but it is unclear which studies report prevalence. | Not clear |
| Goodman, 2014 | Range | 39.00% | 4.40% |  | France, Turkey, USA, Sweden, Nigeria, Italy, Malaysia, Malta | Interviews or scales commonly used and following DSM-IV/ICD-10/Research Diagnostic Criteria. | Mainly recruited from ANC clinics (1 from childbirth preparation course). | 18,525 |
| Sawyer, 2010 | Weighted mean –(aggregate mean, weighted by the number of subjects in each of the contributing studies) | 12.30% | 17.40% | 14.80% | Nigeria | Measures used to screen for disorders varied between studies. The majority of studies conducted structured clinical interviews (n=20), 10 studies used self-administered measures, and 3 studies used both. Anxiety was assessed using the HADS and Self Rating Anxiety Scale (SRAS, Zung, 1971), which are also not diagnostic tools, but have both been validated with African samples. |  | 585 |
| Sharma, 2012 | Range | 1.40% | 0.00% |  | UK, Italy, USA, Sweden | Interviews and self-reported scales (diagnosis based on DSM-IV criteria or Schedule for Affective Disorders and Schizophrenia – Lifetime Version [SADS-L). | Sample size was not clear for 1 study. Population source: 2 studies recruited women attending obstetric services; 1 nationally representative study, and 1 large longitudinal study of pregnant women. | 446 – 1,795** |
| Goodman, 2014 | Range | 10.50% | 0.00% |  | USA, France, Turkey, Sweden, Nigeria, Italy, Malaysia, Brazil, Malta | Interviews or scales commonly used and following DSM-IV/ICD-10/Research Diagnostic Criteria. | Mainly recruited from ANC clinics (1 from childbirth preparation course). | 18,764 |
| Goodman, 2014 | Range | 5.70% | 0.20% |  | USA, France, Turkey, Sweden, Nigeria, Italy, Malaysia, Brazil, Malta, | Interviews or scales commonly used and following DSM-IV/ICD-10/Research Diagnostic Criteria. | Mainly recruited from ANC clinics (1 from childbirth preparation course). | 19,276 |
| Goodman, 2014 | Range | 7.90% | 0.00% |  | France, Turkey, Sweden, Nigeria, Italy, Malaysia, Brazil, USA | Interviews or scales commonly used and following DSM-IV/ICD-10/Research Diagnostic Criteria. | Mainly recruited from ANC clinics (1 from childbirth preparation course). | 10,490 |
| Chico, 2012 | Weighted mean (random effects model) | 36.50% | 22.40% | 29.50% | Malawi, Ethiopia, Sudan, Tanzania, Rwanda, Kenya, Mozambique, Uganda | Periphereal – microscopy based (not molecular). | Population source: Women attending ANC. | 86 – 2,459 |
| Chico, 2012 | Weighted mean (random effects model) | 41.90% | 28.20% | 35.10% | Ghana, Burkina Faso, Cameroon, Nigeria, Mali, Gambia, DRC, Benin | Periphereal – microscopy based (not molecular). | Population source: Women attending ANC. | 38 –,6,370 |
| Chico, 2012 | Weighted mean (random effects model) | 36.40% | 16.70% | 26.50% | Malawi, Sudan, Tanzania, Kenya, Mozambique | Placental – polymerase chain reaction (PCR), Giemsa stain microscopy. | Population source: Women attending ANC. | 85 – 726 |
| Chico, 2012 | Weighted mean (random effects model) | 47.60% | 28.40% | 38.00% | Ghana, Burkina Faso, Nigeria, Cameroon, DRC, Benin | Placental – PCR, Giemsa stain microscopy, antigen assay. | Population source: Women attending ANC. | 36 – 1,875 |
| Merrill, 2011 | Median (weighted according to the quality index score) |  |  | 4.30% | **Algeria, Burkina Faso, Cameroon, Gabon, Ghana, Nigeria, Senegal, Congo, Côte d'Ivoire, Malawi, South Africa, Tanzania, Zambia, Zimbabwe, Canada, US, Belize, Brazil, Mexico, Puerto Rico, Peru, Jordan, Lebanon, Saudi Arabia, Egypt, Pakistan, Croatia, France, Germany, Israel, Italy, Netherlands, Spain, Switzerland, UK, Albania, Bosnia and Herzegovina, Romania, Turkey, Moldavia, Russian Federation, Indonesia, Bangladesh, Bhutan, India, Australia, Brunei, Japan, China, Papua New Guinea, Philippines, Vietnam | Several testing methods: enzyme immunoassay (EIA), reverse passive hemagglutination assay (RPHA), enzyme-linked immunosorbent assay (ELISA), latex agglutination (LATEX), microparticle enzyme immunoassay (MEIA), immunoenzymatic assay (IA), etc. The most common was ELISA. | Countries list, sample size, and diagnostic methods were based on all studies in the systematic review, not specific to the extracted estimate presented here. | 42 – 99,706** |
| Drake, 2014 | Weighted mean (fixed-effect meta-analysis) | 6.10% | 3.30% | 4.70% | Swaziland, South Africa, Malawi, Kenya, Mozambique, Zimbabwe, Uganda, Rwanda, Nigeria, Burkina Faso, Botswana, Tanzania, Zambia | Assays and re-testing. No self-report. |  | 6,789 |
| Mora, 2016 | Weighted mean (random effects model) | 4.28% | 1.46% | 2.51% | Not clear | Not clear |  | Not clear |
| Rao, 2015 | Random effects model | 3.84% | 2.23% | 3.04% | Cameroon, Gabon, Sudan, Ethiopia, Kenya, Benin, Burkina Faso, Côte d'Ivoire, Ghana, Nigeria | Both non-confirmatory screening assays (ELISA, EIA) and confirmatory assays (Recombinant ImmunoBlot Assay [RIBA], western blots, PCR). | Recruitment in ANC clinics. | 19,838 |
| Riou, 2016 | Range | 9.20% | 0.20% |  | Angola, Cameroon, Gabon, Ethiopia, Tanzania, Algeria, Sudan, Tunisia, Zimbabwe, Benin, Burkina Faso, Ghana, Côte d'Ivoire, Mali, Nigeria | The following were included:  (i) rapid test only;  (ii) one ELISA/EIA test only;  (iii) rapid test confirmed by ELISA/EIA test;  (iv) several ELISA/EIA tests; and  (v) use of immunoblot test. |  | 40 – 5,760 |
| Chico, 2012 | Weighted mean (random effects model) | 7.10% | 3.40% | 5.20% | Mozambique, Zambia, Tanzania, Uganda, Botswana, Malawi | Ligase chain reaction (LCR), PCR, EIA cervical swab, or urine. | Population source: Women attending ANC. | 151 – 835 |
| Chico, 2012 | Weighted mean (random effects model) | 3.50% | 0.20% | 1.90% | Ghana, DRC | PCR cervical swab. | Population source: Women attending ANC. | 261 – 521 |
| Joseph Davey, 2016 | Adjusted Mean | 5.60% | 2.80% | 4.20% | **Kenya, Tanzania, Somalia, Ethiopia, Uganda, Sudan | Several lab tests; estimates were adjusted by sensitivity. | Countries list: The list is for all estimates in the paper relevant to Eastern Africa – not specific to syphilis itself. | 856 |
| Joseph Davey, 2016 | Adjusted Mean | 6.60% | 2.30% | 4.40% | **South Africa, Malawi, Madagascar, Zambia, Mozambique, Zimbabwe | Several lab tests; estimates were adjusted by sensitivity. | Countries list: The list is for all estimates in the paper relevant to Eastern Africa – not specific to syphilis itself. | 1,840 |
| Joseph Davey, 2016 | Adjusted Mean | 16.40% | 6.00% | 11.20% | **Peru, Brazil, Ecuador, Argentina, Guatemala | Several lab tests; estimates were adjusted by sensitivity. | Countries list: The list is for all estimates in the paper relevant to Eastern Africa – not specific to syphilis itself. | 4,592 |
| Joseph Davey, 2016 | Adjusted Mean | 1.10% | 0.40% | 0.80% | **China, India, Bangladesh, Papua New Guinea, Turkey, Pakistan, Iran, Myanmar, Cambodia. | Several lab tests; estimates were adjusted by sensitivity. | Countries list: The list is for all estimates in the paper relevant to Eastern Africa – not specific to syphilis itself. | 1,375 |
| Chico, 2012 | Weighted mean (random effects model) | 3.60% | 2.10% | 2.90% | Botswana, Malawi, Mozambique, Zimbabwe, Tanzania, Uganda, Somalia, Ethiopia | Rapid Plasma Reagin (RPR) + [*Treponema pallidum* haemagglutination assay (THPA), or microhemagglutination assay – *Treponema pallidum* (MHA-TP) or Determine™ Syphilis TP)]  or  RPR only  or  Venereal Disease Research Laboratory (VDRL) only. | Population source: Women attending ANC. | 245 – 172,777 |
| Chico, 2012 | Weighted mean (random effects model) | 4.60% | 0.40% | 2.50% | Nigeria, Ghana, Burkina Faso, DRC | RPR + THPA  or  VDRL + THPA. | Population source: Women attending ANC. | 230 – 2,133 |
| Joseph Davey, 2016 | Adjusted Mean | 5.40% | 3.70% | 4.60% | **Kenya, Tanzania, Somalia, Ethiopia, Uganda | Several lab tests; estimates were adjusted by sensitivity. | Countries list: The list is for all estimates in the paper relevant to Eastern Africa – not specific to syphilis itself. | 18,043 |
| Joseph Davey, 2016 | Adjusted Mean | 6.30% | 1.70% | 4.00% | **Benin, Democratic Republic of Congo, Nigeria, Burkina Faso | Several lab tests; estimates were adjusted by sensitivity. | Countries list: The list is for all estimates in the paper relevant to Eastern Africa – not specific to syphilis itself. | 21,481 |
| Joseph Davey, 2016 | Adjusted Mean | 8.30% | 4.70% | 6.50% | **South Africa, Malawi, Madagascar, Zambia, Mozambique, Zimbabwe | Several lab tests; estimates were adjusted by sensitivity. | Countries list: The list is for all estimates in the paper relevant to Eastern Africa – not specific to syphilis itself. | 119,962 |
| Joseph Davey, 2016 | Adjusted Mean | 3.30% | 1.20% | 2.20% | **Peru, Brazil, Ecuador, Argentina, Guatemala | Several lab tests; estimates were adjusted by sensitivity. | Countries list: The list is for all estimates in the paper relevant to Eastern Africa – not specific to syphilis itself. | 196,689 |
| Joseph Davey, 2016 | Adjusted Mean | 1.60% | 0.50% | 1.10% | **China, India, Bangladesh, Papua New Guinea, Turkey, Pakistan, Iran, Myanmar, Cambodia | Several lab tests; estimates were adjusted by sensitivity. | Countries list: The list is for all estimates in the paper relevant to Eastern Africa – not specific to syphilis itself. | 3,107,741 |
| Banura, 2013 | Range | 7.30% | 0.20% |  | Burkina Faso, Tanzania, Malawi, Zambia, Zimbabwe, Uganda | Clinical review |  | 175 – 8,105 |
| Cerruto, 2012 | Range | 31.00% | 3.00% |  | Not clear | Questionnaires, some of which are validated, e.g. ICIQ-SF. At least 1 study did not have clear information on assessment method. | Countries list for specific estimate is not clear but Spain was definitely included. Sample size for 1 study was not clear. The recruitment for some of the studies was carried out within the gynecological departments. | 195 – 43,279** |
| Thom, 2010 | Mean (Mean prevalence was calculated by summing the numerator data – no. of women with urinary incontinence in the subset – and dividing by the sum of the denominator data (all women in the subset). The 95% CI for the mean was calculated using the Wald method) | 36.00% | 32.00% | 33.00% | Sweden, UK, USA, Italy, Canada, Australia, Israel, Turkey, Iran |  | Inclusion criteria for population: "Studies on incontinence in population-based sample defined as from one or more district hospitals or from multiple clinics covering a defined geographic area”. However, 2 countries contributing to the estimates are Turkey and Iran, for which hospital recruitment might not always be entirely appropriate. | Not clear |
| Norhayati, 2015 | Range | 62.00% | 0.10% |  | USA, Australia, Canada, Finland, France, Germany, Israel, Japan, Portugal, Singapore, UAE | This estimate only includes studies which used structured clinical interviews.  (NB: Data are also available from questionnaires only). |  | 70 – 511,422 |
| Norhayati, 2015 | Range | 26.30% | 1.00% |  | India, Morocco, Thailand, Uganda | This estimate only includes studies which used structured clinical interviews.  (NB: Data are also available from questionnaires only). |  | 100 – 544 |
| Norhayati, 2015 | Range | 62.00% | 0.10% |  | USA, Australia, Canada, Finland, France, Germany, Israel, Japan, Portugal, Singapore, UAE, India, Morocco, Thailand, Uganda | This estimate only includes studies which used structured clinical interviews.  (NB: Data are also available from questionnaires only). |  | 70 – 511,422 |
| Parsons, 2011 | Range | 50.00% | 4.90% |  | Nepal, Mongolia, Malaysia, Thailand, China, Indonesia, Bangladesh, India, Vietnam, Iran, Pakistan, Lebanon, Uganda, Zambia, Tunisia, Nigeria, Ethiopia, Morocco, South Africa, Malawi, Zimbabwe, Burkina Faso, Chile, Brazil, Guyana, Barbados, Hungary, Turkey | Varied methods are used across different studies. 24 of the 84 studies used diagnostic interviews to establish the presence of postnatal depression, with the remainder reporting on the number of women scoring over specified thresholds on an inventory for depressive symptoms. The EPDS is the most extensively used measure. The authors note that the different scales appear to result in quite different estimates, even for the same women at the same time point. |  | Not clear |
| Sawyer, 2010 | Weighted mean (aggregate mean, weighted by the number of subjects in each of the contributing studies) | 19.10% | 17.60% | 18.30% | Nigeria, Morocco, Gambia, South Africa, Uganda, Ethiopia | Measures used to screen for disorders varied between studies, including structured clinical interviews (N=11) or only self-administered measures (N=10). For studies using clinical interviews, a 2-stage process was usually adopted whereby a questionnaire was used to identify women above a pre-defined threshold and these women were then interviewed to assess depression. Five different questionnaire measures of postpartum depression were used: the EPDS, HADS, BDI, Zung's SRDS, and the PDQ. |  | 10,093 |
| Schmied, 2013 | Range | 16.00% | 9.00% |  | Australia, New Zealand | A variety of methods of measuring depression were used, including clinical interviews and questionnaires. The most commonly used scale was the EPDS. | Estimate relevant to 6 months postpartum. Cohorts were both recruited from general population and from ANC clinics, but it is unclear which studies report prevalence. | Not clear |
| Goodman, 2016 | Random effects model | 4.58% | 0.66% | 1.78% | Nigeria, Spain, USA, Brazil | Structured Clinical Interview for DSM Disorders (SCID), Mini International Neuropsychiatric Interview (MINI) |  | 13,795 |
| Grekin, 2014 | Weighted mean (mixed-effect model) | 3.90% | 2.50% | 3.10% | Nigeria, Australia, UK, USA, Italy, Canada, France, Netherlands, Norway, Germany, Israel, Sweden, Brazil. | Most studies used self-reported measures, and a few used clinical reviews. The authors did stratify by assessment type, but did not find any notable differences (self-report=3%; clinical assessment=3.6%). |  | 15,637 |
| Goodman, 2016 | Random effects model | 2.76% | 0.09% | 1.66% | Spain, Germany, Brazil, USA | SCID, MINI, Alcohol Use Disorder and Associated Disabilities Interview Schedule-DSM-IV version (AUDADIS-IV) |  | 14,537 |
| Goodman, 2016 | Random effects model | 4.91% | 0.01% | 0.38% | Spain, USA | SCID |  | 11,453 |
| Goodman, 2016 | Random effects model | 13.83% | 5.17% | 8.56% | Australia, Spain, Germany, Brazil, USA | SCID, MINI, AUDADIS-IV, CIDI |  | 5,851 |
| Goodman, 2016 | Random effects model | 6.66% | 1.85% | 3.59% | UK, Spain, Germany, Brazil, USA | Psychiatric Assessment (PAS), SCID, MINI, AUDADIS-IV |  | 14,753 |
| Sawyer, 2010 | Weighted mean (aggregate mean, weighted by the number of subjects in each of the contributing studies) | 15.20% | 12.90% | 14.00% | Nigeria, Ethiopia | Anxiety was assessed using the HADS subscale of anxiety, and Zung's SRAS, which are not diagnostic tools, but have both been validated with African samples. | Authors appear to have counted women from the same study more than once to build the estimates. | 3,502 |
| Drake, 2014 | Weighted mean (fixed-effect meta-analysis) | 4.00% | 1.80% | 2.90% | Rwanda, Zimbabwe, Uganda | Assays and re-testing. No self-report. |  | 14,065 |
| Adler 2013 | Weighted mean (random effects model) | 0.11% | 0.00% | 0.03% | Ethiopia, Gambia, India, Oman, Egypt, Lebanon, Jordan, Turkey, Iran |  |  | 34,505 |
| Cowgill, 2015 | Range | 0.41% | 0.03% |  | Nigeria, Côte d’Ivoire, Mali, Niger, Mauritania, Burkina Faso, Senegal, South Sudan, Malawi |  |  | Not clear |
| Zheng, 2009 | Range | 1.56% | 0.01% |  | Malawi, Burkina Faso, Côte d'Ivoire, Mali, Mauritania, Niger, Senegal | Malawi DHS survey questions on proxy symptoms (not validated). For two studies it just says “interview”, but these are the same papers included in Cowgill (and 1 in Adler) – both of which say a physical exam was performed. Exam is good; unvalidated survey is not. |  | 31,040 |
| Roberts, 2011 | Range | 78.69% | 0.00% |  | Venezuela, Gabon, Nigeria, Brazil, Sudan, Ghana, Tanzania, Peru, Burkina Faso, Malawi, India, Senegal, Mali, Cameroon | Blood smears, placenta smears, Giemsa stain methods, microscopy, histidine-rich protein-2 (HRP-2) rapid diagnostic test (RDT), placenta histology, PCR. No information on 21% of studies. |  | 81 – 8,270 |
| Kourlaba, 2015 | Weighted mean (random effects model) | 0.06% | 0.02% | 0.04% | **UK, Norway, Sudan, Canada, USA, Saudi Arabia, Australia | No information, but assessment methods appropriateness was part of the authors quality criteria. | Population source was unspecified but from the individual paper titles it appears that both population-based and hospital-based study samples are included. In addition, recruitment strategy was part of the quality assessment criteria. | 6,987 – 8,330,927** |
| Meng, 2015 | Weighted mean (random effect model) | 0.04% | 0.02% | 0.03% | China, USA, Australia, Canada, Norway, Japan, Sudan, Saudi Arabia, Sweden, Denmark | 18 studies. 11 of the studies used appropriate diagnostic tools – scans and angiography. 5 used ICD codes (Sweden, USA, Canada, Australia, and China). 1 used Doppler, which alone is not appropriate for pulmonary embolism. 1 had data from 1966 and therefore included clinical symptoms as other diagnostics not available. |  | 15,189,659 |

**References**

Abalos E, Cuesta C, Carroli G, et al. Pre-eclampsia, eclampsia and adverse maternal and perinatal outcomes: a secondary analysis of the World Health Organization Multicountry Survey on Maternal and Newborn Health. *BJOG.* 2014;121:14–24.

Adler AJ, Filippi V, Thomas SL, Ronsmans C. Incidence of severe acute maternal morbidity associated with abortion: a systematic review. *Trop Med Int Health*. 2012;17:177–90.

Adler AJ, Ronsmans C, Calvert C, Filippi V. Estimating the prevalence of obstetric fistula: a systematic review and meta-analysis. *BMC Pregnancy Childbirth*. 2013;13:1–14.

Banura C, Mirembe FM, Orem J, Mbonye AK, Kasasa S, Mbidde EK. Prevalence, incidence and risk factors for anogenital warts in Sub Saharan Africa: a systematic review and meta analysis. *Infect Agent Cancer*. 2013;8:1.

Buckley BS, Harreiter J, Damm P, et al. Gestational diabetes mellitus in Europe: prevalence, current screening practice and barriers to screening. A review. *Diabet Med J Br Diabet Assoc.* 2012;29:844–54.

Calvert C, Thomas SL, Ronsmans C, Wagner KS, Adler AJ, Filippi V. Identifying regional variation in the prevalence of postpartum haemorrhage: a systematic review and meta-analysis. *PLoS One*. 2012;7:e41114.

Carroli G, Cuesta C, Abalos E, Gulmezoglu AM. Epidemiology of postpartum haemorrhage: a systematic review. *Best Pract Res Clin Obstet Gynaecol*. 2008;22:999–1012.

Cerruto MA, D’Elia C, Aloisi A, Fabrello M, Artibani W. Prevalence, incidence and obstetric factors’ impact on female urinary incontinence in Europe: a systematic review. *Urol Int*. 2013;90:1–9.

Cheung WMC, Hawkes A, Ibish S, Weeks AD. The retained placenta: historical and geographical rate variations. *J Obstet Gynaecol.* 2011;31:37–42.

Chico RM, Mayaud P, Ariti C, Mabey D, Ronsmans C, Chandramohan D. Prevalence of malaria and sexually transmitted and reproductive tract infections in pregnancy in sub-Saharan Africa: a systematic review. *JAMA*. 2012;307:2079–86.

Conde-Agudelo A, Romero R. Amniotic fluid embolism: an evidence-based review. *Am J Obstet Gynecol*. 2009;201:445.e1–13.

Cowgill KD, Bishop J, Norgaard AK, Rubens CE, Gravett MG. Obstetric fistula in low-resource countries: an under-valued and under-studied problem – systematic review of its incidence, prevalence, and association with stillbirth. *BMC Pregnancy Childbirth*. 2015;15:193.

Cresswell JA, Ronsmans C, Calvert C, Filippi V. Prevalence of placenta praevia by world region: a systematic review and meta-analysis. *Trop Med Int Health.* 2013;18:712–24.

Drake AL, Wagner A, Richardson B, John-Stewart G. Incident HIV during pregnancy and postpartum and risk of mother-to-child HIV transmission: a systematic review and meta-analysis. *PLoS Med*. 2014;11:e1001608.

Einarson TR, Piwko C, Koren G. Quantifying the global rates of nausea and vomiting of pregnancy: a meta analysis. *J Popul Ther Clin Pharmacol.* 2013;20:e171–83.

Frati P, Foldes-Papp Z, Zaami S, Busardo FP. Amniotic fluid embolism: what level of scientific evidence can be drawn? A systematic review. *Curr Pharm Biotechnol.* 2014;14:1157–62.

Goodman JH, Chenausky KL, Freeman MP. Anxiety disorders during pregnancy: a systematic review. *J Clin Psychiatry*. 2014;75:e1153–84.

Goodman JH, Watson GR, Stubbs B. Anxiety disorders in postpartum women: a systematic review and meta-analysis. *J Affect Disord.* 2016;203:292–331.

Grekin R, O’Hara MW. Prevalence and risk factors of postpartum posttraumatic stress disorder: a meta-analysis. *Clin Psychol Rev.* 2014;34:389–401.

Han A, Stewart DE. Maternal and fetal outcomes of intimate partner violence associated with pregnancy in the Latin American and Caribbean region. *Int J Gynecol Obstet.* 2014;124:6–11.

Hirst JE, Raynes-Greenow CH, Jeffery HE. A systematic review of trends of gestational diabetes mellitus in Asia. *J Diabetol*. 2012;3:5.

Hunt KJ, Schuller KL. The increasing prevalence of diabetes in pregnancy. *Obstet Gynecol Clin North Am*. 2007;34:173–99.

Joseph Davey DL, Shull HI, Billings JD, Wang D, Adachi K, Klausner JD. Prevalence of curable sexually transmitted infections in pregnant women in low- and middle-income countries from 2010 to 2015: a systematic review. *Sex Transm Dis*. 2016;43:450–8.

Kanguru L, Bezawada N, Hussein J, Bell J. The burden of diabetes mellitus during pregnancy in low- and middle-income countries: a systematic review. *Glob Health Action*. 2014;7.

Kourlaba G, Relakis J, Kontodimas S, Holm MV, Maniadakis N. A systematic review and meta-analysis of the epidemiology and burden of venous thromboembolism among pregnant women. *Int J Gynecol Obstet.* 2016;132:4–10.

Liepe K, Blättner B. Violence during pregnancy: prevalence studies in OECD countries [in German]. *Gesundheitswesen*. 2013;75:473–80.

Macaulay S, Dunger DB, Norris SA. Gestational diabetes mellitus in Africa: a systematic review. *PLoS One*. 2014;9:e97871.

Mendez-Figueroa H, Dahlke JD, Vrees RA, Rouse DJ. Trauma in pregnancy: an updated systematic review. *Am J Obstet Gynecol.* 2013;209:1–10.

Meng K, Hu X, Peng X, Zhang Z. Incidence of venous thromboembolism during pregnancy and the puerperium: a systematic review and meta-analysis. *J Matern Fetal Neonatal Med.* 2015;28:245–53.

Merrill RM, Hunter BD. Seroprevalence of markers for hepatitis B viral infection. *Int J Infect Dis.* 2011;15:e78–121.

Mora N, Adams WH, Kliethermes S, et al. A synthesis of hepatitis C prevalence estimates in Sub-Saharan Africa: 2000–2013. *BMC Infect Dis*. 2016;16:283.

Mwanri AW, Kinabo J, Ramaiya K, Feskens EJM. Gestational diabetes mellitus in sub-Saharan Africa: systematic review and metaregression on prevalence and risk factors. *Trop Med Int Health*. 2015;20:983–1002.

Norhayati MN, Hazlina NH, Asrenee AR, Emilin WM. Magnitude and risk factors for postpartum symptoms: a literature review. *J Affect Disord*. 2015;175:34–52.

Padua L, Di Pasquale A, Pazzaglia C, Liotta GA, Librante A, Mondelli M. Systematic review of pregnancy-related carpal tunnel syndrome. *Muscle Nerve*. 2010;42:697–702.

Parsons C, Young K, Rochat T, Kringelbach M, Stein A. Postnatal depression and its effects on child development: a review of evidence from low- and middle-income countries. *Br Med Bull*. 2012;101:57–79.

Puccia MI, Mamede MV. Integrative review regarding intimate partner violence in pregnancy [in Portuguese]. Rev Eletr Enf. 2012;14:944–56.

Rao VB, Johari N, du Cros P, Messina J, Ford N, Cooke GS. Hepatitis C seroprevalence and HIV co-infection in sub-Saharan Africa: a systematic review and meta-analysis. *Lancet Infect Dis*. 2015;15:819–24.

Riou J, Aït Ahmed M, Blake A, et al. Hepatitis C virus seroprevalence in adults in Africa: a systematic review and meta-analysis. *J Viral Hepat*. 2016;23:244–55.

Roberts T, Gravett CA, Velu PP, et al. Epidemiology and aetiology of maternal parasitic infections in low- and middle-income countries. *J Glob Health*. 2011;1:189–200.

Sangsawang B, Sangsawang N. Stress urinary incontinence in pregnant women: a review of prevalence, pathophysiology, and treatment. *Int Urogynecology J*. 2013;24:901–12.

Sawyer A, Ayers S, Smith H. Pre- and postnatal psychological wellbeing in Africa: a systematic review. *J Affect Disord*. 2010;123:17–29.

Schmied V, Johnson M, Naidoo N, et al. Maternal mental health in Australia and New Zealand: a review of longitudinal studies. *Women Birth J Aust Coll Midwives.* 2013;26:167–78.

Schneider S, Bock C, Wetzel M, Maul H, Loerbroks A. The prevalence of gestational diabetes in advanced economies. *J Perinat Med*. 2012;40:511–20.

Shamu S, Abrahams N, Temmerman M, Musekiwa A, Zarowsky C. A systematic review of African studies on intimate partner violence against pregnant women: prevalence and risk factors. *PLoS One*. 2011;6:e17591.

Sharma V, Pope CJ. Pregnancy and bipolar disorder: a systematic review. *J Clin Psychiatry*. 2012;73:1447–55.

Thom DH, Rortveit G. Prevalence of postpartum urinary incontinence: a systematic review. *Acta Obstet Gynecol Scand*. 2010;89:1511–22.

Villot A, Deffieux X, Demoulin G, Rivain A-L, Trichot C, Thubert T. Management of third and fourth degree perineal tears: a systematic review [in French]. *J Gynecol Obstet Biol Reprod (Paris).* 2015;44:802–11.

Zheng AX, Anderson FW. Obstetric fistula in low-income countries. *Int J Gynecol Obstet*. 2009;104:85–9.

Zhu Y, Zhang C. Prevalence of gestational diabetes and risk of progression to Type 2 diabetes: a global perspective. *Curr Diab Rep*. 2016;16:7.
