# Supplementary material for: The frequency of maternal morbidity: A systematic review of systematic reviews
Source: Int J Gynaecol Obstet. 2018 May 23;141(Suppl Suppl 1):20–38. doi: 10.1002/ijgo.12468 (PMC6001670; doi:10.1002/ijgo.12468)
Supplement: Supplementary file 5 — Appendix S5. Timing of postpartum depression assessment. [file IJGO-141-20-s005.docx]

**Appendix S5.** Timing of postpartum depression assessment.

| Study | Region | Inclusion criteria for overall estimates presented by the systematic review | Estimates for postpartum depression (PPD) at different time points postpartum |
| --- | --- | --- | --- |
| Norhayati, 2015^1^ | Low- and middle-income settings | No inclusion criteria stated for selecting an estimate from studies with data at multiple time points. | Ranges are reported for the prevalence of PPD stratified by time of assessment:  <4 weeks: 12.9–50.7%  4–8 weeks: 4.9–50.8%  6 months: 6.6–38.2%  First year: 6.7–57.2% |
| Parsons, 2011^2^ | Low- and middle-income settings | The earliest time point between 1 week and 1 year postpartum was selected from studies with multiple time points. | No information. |
| Sawyer, 2010^3^ | Africa | Not clear how estimates were selected for inclusion where multiple estimates per paper were available. | Authors note that longitudinal studies show that the prevalence of PPD drops off after six months. |
| Schmied, 2013^4^ | Australia and New Zealand | No information. | Reported prevalence of PPD at different time points for a primary study:  3 months postpartum: 7.3%  6 months postpartum: 9.1%  6–9 months postpartum: 9.5% |

**References**

[1] Norhayati MN, Hazlina NHN, Asrenee AR, Emilin WMAW. Magnitude and risk factors for postpartum symptoms: a literature review. *J Affect Disord*. 2015;175:34–52. doi:10.1016/j.jad.2014.12.041.

[2] Parsons C, Young K, Rochat T, Kringelbach M, Stein A. Postnatal depression and its effects on child development: a review of evidence from low- and middle-income countries. *Br Med Bull*. 2012;101:57–79.

[3] Sawyer A, Ayers S, Smith H. Pre- and postnatal psychological wellbeing in Africa: a systematic review. *J Affect Disord*. 2010;123:17–29. doi:10.1016/j.jad.2009.06.027.

[4] Schmied V, Johnson M, Naidoo N et al. Maternal mental health in Australia and New Zealand: a review of longitudinal studies. *Women Birth J Aust Coll Midwives.* 2013;26:167–178. doi:10.1016/j.wombi.2013.02.006.
